# Mechanism-guided drug development and treatment for liver fibrosis: a clinical perspective

**DOI:** 10.3389/fphar.2025.1574385

**Published:** 2025-05-26

**Authors:** Xiangchang Zeng, Deliang Huang, Zhibin Zhu, Qingxian Cai, Yang Yang, Hongzhou Lu, Jun Chen

**Affiliations:** ^1^ Department of Liver Diseases, Shenzhen Third People's Hospital, The Second Affiliated Hospital of Southern University of Science and Technology, Shenzhen, Guangdong, China; ^2^ Shenzhen Key Laboratory of Pathogen and Immunity, Shenzhen Clinical Research Center for Infectious Disease, State Key Discipline of Infectious Disease, Shenzhen Third People's Hospital, The Second Hospital Affiliated to Southern University of Science and Technology, Shenzhen, Guangdong, China

**Keywords:** liver fibrosis, pathogenesis, anti-fibrotic drug, mechanism-guided drug development and treatment, clinical study

## Abstract

Liver fibrosis is a common response to chronic liver injury due to multiple etiologies and plays a crucial in the progression of chronic liver disease to cirrhosis, hepatocellular carcinoma, and other liver-related clinical outcomes. Currently, available treatments to block liver fibrosis are designed to eliminate the underlying causes of liver disease. The lack of truly effective drugs to regress or reverse fibrosis is a major unmet clinical need. In this context, this article briefly describes the pathological process of hepatic fibrosis and focuses on reviewing the progress of clinical studies on mechanism-based anti-fibrotic drug development and therapy, highlighting that the positive effect of thyroid hormone receptor-β (THR-β) analogs, fibroblast growth factor 21 (FGF21) analogues, Glucagon-like peptide 1 receptor (GLP-1R) agonists, pan-peroxisome proliferator-activated receptor (pan-PPAR) agonists, fatty acid synthase (FASN) inhibitors, and hydronidone in reducing liver fibrosis caused by specific etiologies. Moreover, multi-pathway guided combination therapy or traditional Chinese medicine demonstrate significant advantages in combating liver fibrosis. Finally, new technologies and approaches affecting the clinical development of anti-hepatic fibrosis drugs were discussed.

## 1 Introduction

Liver fibrosis is a common pathological process in chronic liver diseases caused by various etiologies, including hepatitis virus infections, metabolic and genetic diseases, autoimmune conditions, and drug-induced insults (Böttcher and Pinzani, 2017). Liver fibrosis is characterized by abnormal or excessive deposition of extracellular matrix (ECM) due to an uncontrolled wound healing response following chronic liver injury (Roehlen et al., 2020).

Numerous evidences showed that hepatic fibrosis without effective treatment will progress to cirrhosis, hepatocellular carcinoma, and even liver-related death (Sun Y. et al., 2024; Vilar-Gomez et al., 2018; Angulo et al., 2015). Therefore, the development of effective therapeutic strategies to reverse or delay liver fibrosis has become a key clinical issue in the treatment of chronic liver diseases.

Eliminating or controlling the etiology of chronic liver injury such as viruses, nonalcoholic fatty liver disease (NAFLD), and autoimmune liver diseases, etc., can lead to regression of liver fibrosis (Trautwein et al., 2015; Lee et al., 2015). However, many patients do not respond to treatment, which results in advanced liver fibrosis or cirrhosis. Currently, available specific anti-liver fibrosis drugs are limited (Khanam et al., 2021). Recently, a deeper understanding of the molecular and cellular mechanisms underlying the development of liver fibrosis has led to the identification of several potential therapeutic targets (Akkız et al., 2024), and drugs based on these targets have entered clinical studies (Roehlen et al., 2020), some of which have achieved positive results.

This article summarizes the pathological process and clinical research progress of mechanism-based anti-liver fibrosis drugs to provide guidance for the orderly development of drugs for the prevention and treatment of liver fibrosis in the future, thereby benefiting patients with chronic liver disease.

## 2 The process and pathogenesis of liver fibrosis

**FIGURE 1:**
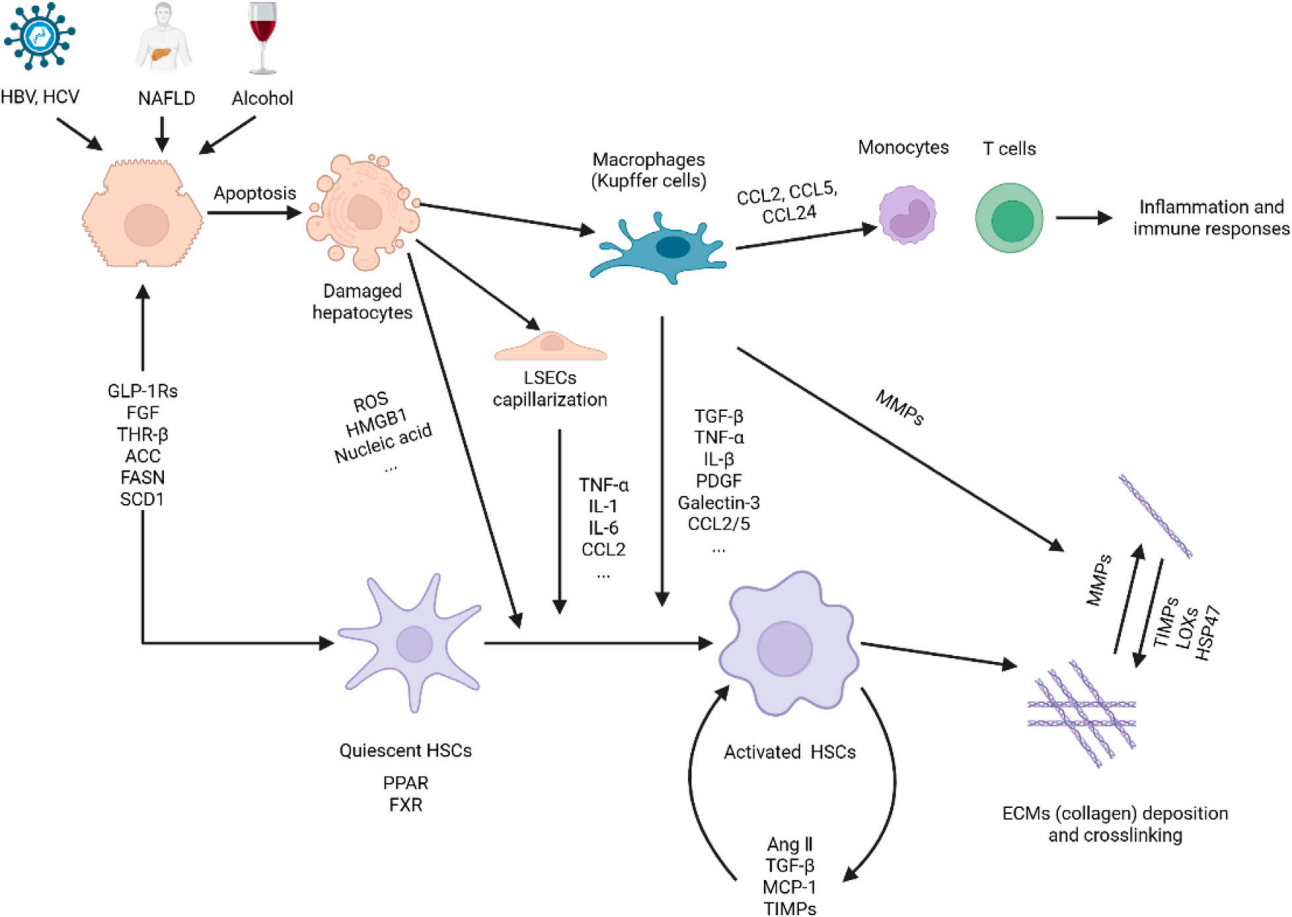
Pathogenesis of liver fibrosis. Hepatocytes are damaged under the stimulation of viruses, NAFLD, alcohol, etc., releasing reactive oxygen species, nucleic acids, and HMGB1 to activate immune cells such as macrophages (Kupffer cells), liver sinusoidal endothelial cells (LSECs) and HSCs. On the one hand, activated macrophages secrete cytokines and chemokines to establish a fibrogenic environment, induce HSCs activation. On the other hand, macrophages release MMPs to promote the degradation of extracellular matrix, thereby facilitating the resolution of fibrosis. Activated HSCs were transdifferentiated into myofibroblast-like cells, which not only secrete cytokines, growth factors, and chemokines to interact with macrophages to further induce HSCs activation, but also induce the synthesis and secretion of extracellular matrix. HSC activation is not only affected by the above-mentioned cytokines and chemokines, but also regulated by glycolipid metabolism and related and nuclear receptors. Finally, the imbalance between extracellular matrix synthesis and degradation leads to excessive deposition and cross-linking of extracellular matrix to form fibrous scars. This figure was adapted from the literature (Zoubek et al., 2017; Horn and Tacke, 2024; Bataller and Brenner, 2005; Campana and Iredale, 2017; Zhao M. et al., 2022; Tacke et al., 2023).

The formation and progression of liver fibrosis is a complex process that includes liver injury, an inflammatory cascade, activation of hepatic stellate cells (HSCs), and formation of fiber scar caused by extracellular matrix deposition (Figure 1).

Harmful substances, such as hepatitis B (HBV) or hepatitis C (HCV) virus infections, NAFLD, autoimmune liver diseases, and alcohol, persistently stimulate hepatocyte or cholangiocyte death through necrosis and apoptosis, which are the initiating events of liver fibrosis (Lee et al., 2015). Damaged hepatocytes release reactive oxygen species and damage-associated molecular patterns (DAMPs), such as nucleic acids and high-mobility group box 1, which in turn further cause hepatocyte injury, inflammatory responses and directly induce the activation of hepatic stellate cells (HSCs) (Zoubek et al., 2017).

During chronic injury, Kupffer cells (hepatic macrophages) are activated by DAMPs, lipopolysaccharides or viral DNA to elicit the secretion of many cytokines (TNF-α, IL-1β, IL-6, TGF-β, PDGF, and galectin-3) and chemokines (CCL2, CCL5, and CCL24) (Parola and Pinzani, 2019), creating a fibrotic microenvironment to mediate pro-inflammatory responses and the activation of HSCs (Hammerich and Tacke, 2023). Additionally, these chemokines can recruit monocytes and T cells to enter the injured liver for activation and proliferation, and further generate an inflammatory environment (Trautwein et al., 2015). Conversely, specific macrophages can protect the liver by releasing anti-inflammatory cytokines IL-10 and matrix metalloproteinases (MMPs), including MMP9, MMP12, and MMP13, to resolve fibrosis (Cheng et al., 2021).

Liver sinusoidal endothelial cells (LSECs) are gatekeepers that maintain HSCs quiescence by releasing NO, and provide anti-inflammatory signals leading to immune tolerance. However, under the stimulation of metabolic NASH, LPS, and other chronic injuries, LSECs loses its differentiation phenotype also known as LSECs capillarization. The dedifferentiated LSECs induces HSC activation, and also secretes a series of cytokines and chemokines (TNF-α, IL-6, IL-1, CCL2) to generate a hepatic inflammatory environment further inducing fibrogenesis (Gilgenkrantz et al., 2025). Furthermore, the dedifferentiated LSECs can also secrete profibrogenic molecules such as transforming growth factor-β (TGF-β), fibronectin, laminin, VAP-1 and adhesion molecules (ICAM-1, VCAM-1) to directly activate HSCs to accelerate the progression of liver fibrosis. Dysfunction of LSECs can disrupt their communication with HSCs, Kupffer cells and hepatocytes, causing homeostasis imbalance and thereby promoting the formation of liver fibrosis (Dai et al., 2025; Qu et al., 2024). Collectively, LSECs are involved in hepatic fibrogenesis through multiple pathways and are gradually being recognized as important players in liver fibrosis.

HSCs are the primary cells for the production of the extracellular matrix located in the gap between hepatocytes and sinusoidal endothelial cells. Under normal conditions, HSCs in the resting state store vitamin A and regulate sinusoidal blood flow. In chronic liver injury, quiescent HSCs are activated and transdifferentiated into myofibroblast-like cells (Trautwein et al., 2015). Activated HSCs migrate into the injured liver to synthesize or secrete extracellular matrix directly or inhibit extracellular matrix degradation by releasing tissue inhibitor of metalloproteinases (TIMPs), which is critical in hepatic fibrosis and fibrous scar formation (Tsuchida and Friedman, 2017). Thus, understanding the fate of HSCs is key to elucidating the pathogenesis of liver fibrosis (Higashi et al., 2017).

**FIGURE 2:**
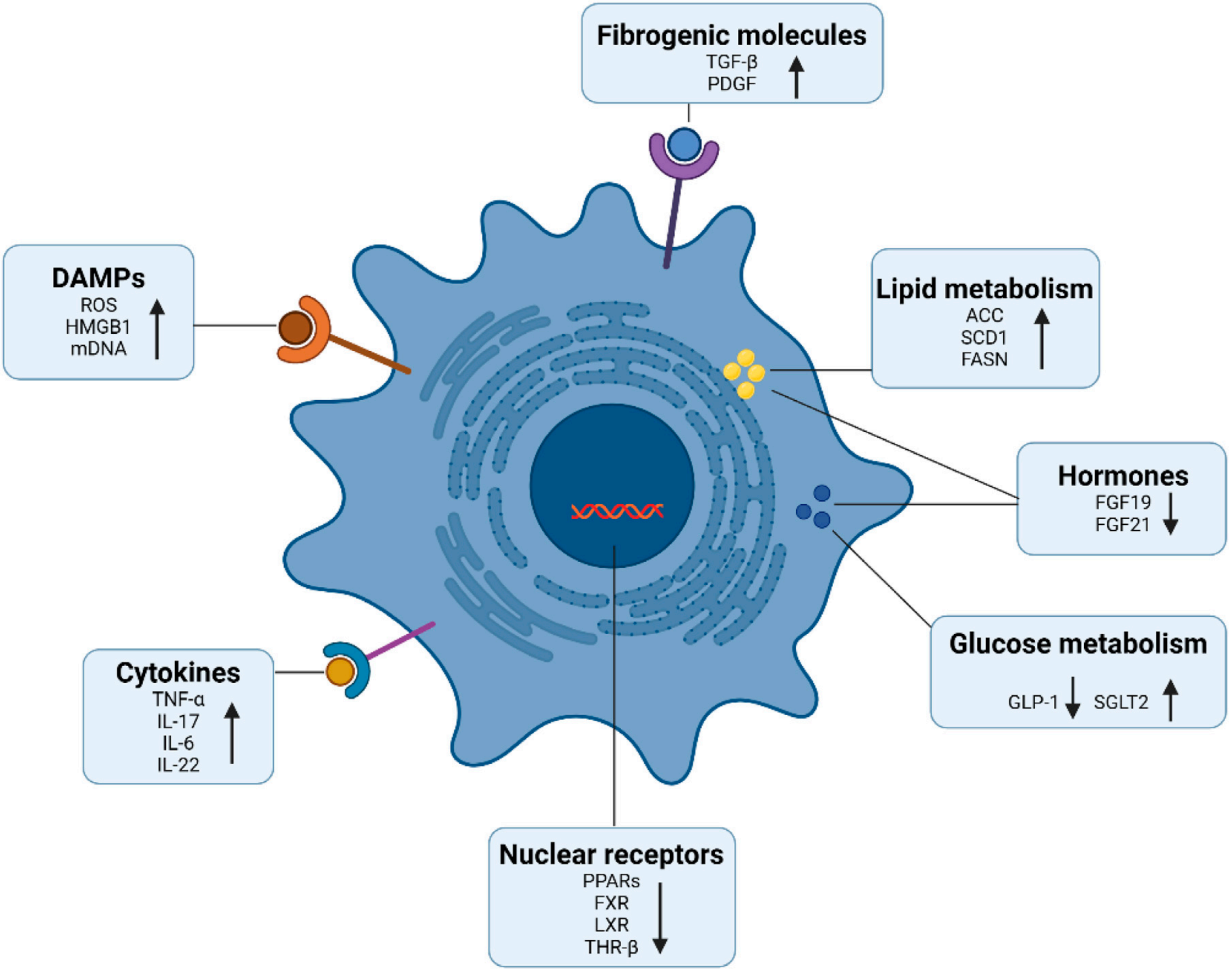
Signaling molecules involved in HSCs activation. A series of signaling molecules regulate the activation of hepatic stellate cells, such as damage-associated molecular patterns, cytokines, and pro-fibrotic growth factors. In addition, activated HSCs obtain energy through glucose and lipid metabolism. Moreover, nuclear receptors not only modulate cellular metabolism, but also modulate HSCs activation and the expression of extracellular matrix including collagen. This figure was adapted from the literature (Tsuchida and Friedman, 2017; Horn and Tacke, 2024; Tacke et al., 2023).

Considerable progress has been made in uncovering molecules that regulate the proliferation and activation of HSCs (Figure 2). Studies have shown that many cytokines can promote the activation of HSCs, among which TGF-β has the strongest effect, driving cell activation and collagen expression through smad2/3-dependent or independent pathways (Kisseleva and Brenner, 2021). Activated HSCs can also release angiotensin II, MCP-1, TGF-β, and reactive oxygen species in an autocrine manner, interacting with immune cells to form a fibrogenic environment that further promotes HSCs proliferation and activation (Cheng et al., 2021; Tsuchida and Friedman, 2017). In recent years, glucose, lipid, and bile acid metabolism signals (FGF, THR-β, FSAN, ACC, and SCD1) and related nuclear receptors (PPARs, FXRs) have been shown to regulate the activation of HSCs and the synthesis of their fibrosis-related proteins (Horn and Tacke, 2024).

Fibrogenesis is the terminal event in the formation of fibrous scarring, which is a process in which activated HSCs release extracellular matrix molecules that gradually mature and cross-link into a mesh. These processes are influenced by tissue inhibitor of metalloproteinases (TIMPs), heat shock protein 47 (HSP47) (Abd El-Fattah and Zakaria, 2022), and lysyl oxidase 2 (LOXL2) (Chen et al., 2020). MMPs released from macrophages and HSCs degrade the extracellular matrix, including type I collagen, and are involved in the regression of fibrosis (Roeb, 2018). Generally, fibrous scars can only be formed when the synthesis and degradation of the extracellular matrix are imbalanced, resulting in excessive deposition.

## 3 Target-mediated anti-liver fibrosis drugs

With the growing understanding of the molecular and cellular mechanisms of liver fibrosis, numerous targets have emerged and driven the development of anti-hepatic fibrotic drugs. We searched the clinical studies on anti-hepatic fibrosis drugs over the past 5 years and found that current development strategies focus on metabolic regulation, apoptosis and inflammation, proliferation and activation of HSCs, and synthesis and degradation of the extracellular matrix. This section reviews the progress of clinical research on anti-fibrotic drugs guided by targets derived from these pathways. The main clinical outcomes of these drugs are summarized in Table 1.

**TABLE 1:** Clinical outcomes of target-based drug development for liver fibrosis.

Target	Drugs	Etiology	Dosage	Duration of administration	Sample size	Blood markers of fibrosis	Imaging biomarkers of fibrosis	Histological improvement in fibrosis	Clinical trial (phase)	References
Drugs that regulate metabolism										
GLP-1R	Semaglutide	NASH and moderate to advanced liver fibrosis (stage 2 or 3)	2.4 mg	72 weeks	800	Unknown	Unknown	37.0% of people treated with semaglutide achieved improvement in liver fibrosis with no worsening of steatohepatitis compared to 22.5% on placebo. 62.9% of people treated with semaglutide achieved resolution of steatohepatitis3 with no worsening of liver fibrosis compared to 34.1% on placebo.	3	Novo Nordisk official website
	Semaglutide	NASH with cirrhosis	2·4 mg	48 weeks	71	Pro-C3	Negative	Negative	2	Loomba et al. (2023a)
GIP/GLP-1R	Tirzepatide	NASH and stage F2 or F3 fibrosis	5, 10, 15 mg	52 weeks	190	Pro-C3, Enhanced Liver Fibrosis test score	A trend of improvement	A trend of improvement but no significant	2	Loomba et al. (2024a)
GCG/GLP-1R	Survodutide	NASH and fibrosis stage F1 through F3	6.0 mg	48 weeks	295	Unknown	Unknown	50.0% of participants had ≥1 stage improvement in fibrosis without worsening of NASH in the 6.0 mg survodutide group compared with 21.2% with placebo (p < 0.001).	2	Sanyal et al. (2024b)
GCGR/GLP-1	Cotadutide	Noncirrhotic NASH with fibrosis	300, 600 μg	19 weeks	74	Pro-C3, AST-to-Platelet Ratio Index	Negative	Negative	2	Shankar et al. (2024)
	Cotadutide	Overweight or obesity and type 2 diabetes	100, 200, 300 μg	54 weeks	834	FIB-4, Pro-C3, NAFLD fibrosis score	Unknown	Unknown	2	Nahra et al. (2021)
FGF19	Aldafermin	NASH and compensated cirrhosis	0.3, 1, 3 mg	48 weeks	160	Pro-C3, Enhanced liver fibrosis	Negative	Negative	2	Rinella et al. (2024)
	Aldafermin	NASH and stage 2 or 3 fibrosis	0.3, 1, 3 mg	24 weeks	171	Pro-C3, Enhanced liver fibrosis	Negative	Negative	2	Harrison et al. (2022)
FGF21	Pegbelfermin	NASH and compensated cirrhosis	10, 20, 40 mg	48 weeks	155	Negative	Negative	Negative	2	Abdelmalek et al. (2024)
	Pegozafermin	NASH and stage F2 or F3 fibrosis	15, 30, 44 mg	24 weeks	222	Pro-C3, Enhanced liver fibrosis, FIB-4,	Positive	The percentage of patients achieved fibrosis improvement was 7% in the placebo group, 22% in the 15 mg pegozafermin group, 26% in the 30 mg pegozafermin group (P = 0.009), and 27% in the 44-mg pegozafermin group (P = 0.008).	2	Loomba et al. (2023c)
	Efruxifermin	NASH and stage F2 or F3 fibrosis	28, 50 mg	24 weeks	128	Pro-C3, Enhanced liver fibrosis	Positive	20% of patients in the placebo group had an improvement in fibrosis of at least 1 stage and no worsening of NASH versus 39% of patients in the efruxifermin 28 mg group (p = 0.025) and 41% of patients in the efruxifermin 50 mg group (p = 0.036).	2	Harrison et al. (2023b)
ACC	Firsocostat (GS-0976, NDI-010976)	NASH and F1-F3 fibrosis	5, 20 mg	12 weeks	126	TIMP-1	Negative	Negative	2	Loomba et al. (2018a)
FASN	Denifanstat (TVB-2640)	NASH and stage F2 or F3 fibrosis	50 mg	52 weeks	168	Negative	Negative	Improvement of fibrosis by one stage or more without worsening of steatohepatitis was attained in 41% of participants in the denifanstat group compared with 18% of participants in the placebo group (p = 0.0102).	2	Loomba et al. (2024c)
SCD1	Aramchol	NASH	400, 600 mg	52 weeks	247	FIB-4, NAFLD Fibrosis Scores	Unknown	Fibrosis improvement by ≥1 stage without worsening NASH was achieved in 29.5% of aramchol 600 mg versus 17.5% of the placebo arm (p = 0.21).	2	Ratziu et al. (2021)
FFAR1/FFAR4	Icosabutate	NASH with liver fibrosis	300, 600 mg	52 weeks	187	Enhanced liver fibrosis test score	Unknown	A higher proportion of patients treated with icosabutate 300 mg achieved an improvement in fibrosis of at least one stage, with responder rates of 11.3%, 29.3% and 23.9% in the placebo, 300 mg and 600 mg treatment groups, respectively.	2	Harrison et al. (2025a)
THR-β	Resmetirom^a^	NASH with liver fibrosis	80, 100 mg	52 weeks	966	Enhanced liver fibrosis test score	Negative	Fibrosis improvement by at least one stage with no worsening of the NAFLD activity score was achieved in 24.2% of the patients in the 80-mg resmetirom group and 25.9% of those in the 100 mg resmetirom group, as compared with 14.2% of those in the placebo group (p < 0.001).	3	Harrison et al. (2024b)
Pan-PPAR	Lanifibranor	NASH	800, 1,200 mg	24 weeks	247	Negative	Negative	The 1200-mg and 800-mg doses of lanifibranor over placebo for improvement in fibrosis stage of at least 1 without worsening of NASH (48% and 34%, respectively, vs. 29%)	2	Francque et al. (2021)
FXR	Obeticholic acid	NASH with stage F2-F3 fibrosis	10, 25 mg	18 months	931	Unknown	Unknown	The fibrosis improvement endpoint was achieved by 12% patients in the placebo group, 18% in the obeticholic acid 10 mg group (p = 0.045), and 23% in the obeticholic acid 25 mg group (p = 0.0002).	3	Younossi et al. (2019)
Drugs targeting apoptotic signaling										
Caspase	Emricasan	NASH and F1-F3 fibrosis	5, 50 mg	72 weeks	318	Unknown	Unknown	Negative	2	Harrison et al. (2020b)
ASK1	Selonsertib	NASH and bridging fibrosis or compensated cirrhosis	6, 18 mg	48 weeks	320	Negative	Negative	Negative	3	Harrison et al. (2020c)
Inflammatory and immune response modulators										
CCR2/5	Cenicriviroc	NASH and stage 2 or 3 liver fibrosis	150 mg	12 months or 60 months	1,293 or 485	Negative	Negative	Negative	3	Anstee et al. (2024)
CCL24	CM101	NAFLD	2.5, 5 mg/kg	12 weeks	10	TIMP1, TIMP2, PDGF-AA	Unknown	Unknown	1	Mor et al. (2024)
PDE	ZSP1601	NAFLD	50, 100 mg	28 days	36	FAST score	Positive	Unknown	1	Hu et al. (2023)
VAP-1	Timolumab	Primary sclerosing cholangitis	8 mg/kg	99 days	23	Negative	Unknown	Unknown	2	Hirschfield et al. (2024b)
Galectin-3	Belapectin (GR-MD-02)	NASH with cirrhosis and portal hypertension	2, 8 mg/kg	52 weeks	162	Negative	Unknown	Negative	2	Chalasani et al. (2020)
Drugs targeting the activation of hepatic stellate cells										
TGF-β	Pirfenidone	Advanced liver fibrosis	600 mg	12 months	281	TGF-β1	Positive	Unknown	2	Poo et al. (2020)
	Hydronidone	Chronic hepatitis B-associated liver fibrosis	180, 270, 360 mg	52 weeks	168	Unknown	Unknown	The fibrosis improvement endpoint was achieved by 25.6% of patients in the placebo group and 40.5% of patients in the 180-mg group (P = 0.12), 54.8% of patients in the 270-mg group (P = 0.006), and 43.90% of patients in the 360-mg group (P = 0.08). The improvement rate was 46.4% in the combined hydronidone group (p = 0.014).	2	Cai et al. (2023)
Angiotensin II type 1 receptor	Candesartan	Compensated alcoholic liver fibrosis	8 mg	6 months	85	α-smooth muscle actin, hydroxyproline, TGF-β1, collagen-1, TIMP-1, MMP2	Unknown	Candesartan showed significantly higher rates of histological improvements (33.3% vs. 11.6%, p = 0.020).	—	Kim et al. (2012)
Drugs targeting extracellular matrix synthesis and degradation										
HSP47	BMS-986263 (ND-L02-s0201)	Hepatic fibrosis secondary to HCV infection	45, 90 mg	12 weeks	61	A tendency to improve.	A tendency to improve.	13% in placebo, 17% in 45 mg, and 21% in 90 mg had METAVIR improvements of ≥1 stage.	2	Lawitz et al. (2022)
LOXL2	Simtuzumab	NASH with bridging fibrosis or compensated cirrhosis	75, 125 mg for bridging fibrosis, 200 or 700 mg for compensated cirrhosis	96 weeks	219/258	Negative	Unknown	Negative	2	Harrison et al. (2018c)
Chinese herbal compound prescription										
Multi-target and pathway	Biejia-Ruangan compound (BRC)	Chronic Hepatitis B with advanced fibrosis or cirrhosis	2 g	72 weeks or 7 years of follow-up	500 or 1,000	Unknown	Unknown	The rate of fibrosis regression and the rate of cirrhosis reversal were significantly higher in the Biejia-Ruangan plus entecavir group than in the placebo group, respectively (40% vs. 31.8%, p = 0.0069 or 41.5% vs. 30.7%, p = 0.0103). The combination treatment reduced the risk of hepatocellular carcinoma and liver-related deaths.	4	Rong et al. (2022), Ji et al. (2022)
Multi-target and pathway	Fuzheng Huayu formula (FZHY)	Chronic hepatitis B patients with Ishak fibrosis score ≥ 3	1.6 g FZHY three times daily	48 weeks	52	Unknown	Unknown	The proportion of the CHB patients with Ishak fibrosis in the combination group with at least a 1-point improvement was significantly higher than that in the control group (81.8% vs. 54.2%, p < 0.05).	Pilot study	Gui et al. (2020)
		Patients with chronic hepatitis C	4.8 g/day	48 weeks	118	Unknown	Unknown	The FZHY-treated group with baseline Ishak fibrosis stages F3 and F4 had a better response than patients with baseline Ishak fibrosis stages F0–F2 (p = 0.03).	2	Hassanein et al. (2022)
Multi-target and pathway	AnluoHuaxian pill (AHP)	Chronic hepatitis B patients with advanced fibrosis or cirrhosis	6 g	48 weeks	270	Unknown	Positive	The rate of histologic improvement in liver fibrosis patients in the AHP group was significantly higher than that in the placebo group (37.7% vs. 19.5%, p = 0.035)	4	Xiao et al. (2022)
Multi-target and pathway	Ruangan granule (RG)	Chronic hepatitis B patients with advanced fibrosis or cirrhosis	Two times/day	48 weeks or 55 months of follow up	240	FIB-4	Positive	The rate of fibrosis regression and inflammation remission in histopathology was significantly higher in the entecavir + RG group than that of the control (entecavir) group (38.73% vs. 23.94%, p = 0.031). The combination treatment reduced the risk of hepatocellular carcinoma.	4	Xing et al. (2023)

^a^ This drug was approved by the U.S., Food and Drug Administration for the treatment of NASH-related liver fibrosis. Unknown represents unmeasured or lack of data in this study.

### 3.1 Drugs that regulate metabolism

#### 3.1.1 Glucagon-like peptide 1 receptor (GLP-1R) agonist

Semaglutide is a long-acting glucagon-like peptide 1 receptor (GLP-1R) agonist approved for treating type 2 diabetes and obesity. Due to its lipid-lowering and anti-inflammatory effects, studies have examined the effects of semaglutide on NASH and found that semaglutide increased the percentage of patients who achieved resolution of NASH with no worsening of liver fibrosis as well as noninvasive fibrosis biomarkers, but had no significant effect on fibrosis improvement by histological evaluation (Newsome et al., 2021; Ratziu et al., 2024a). In patients with NASH and compensated cirrhosis, researchers also observed that semaglutide did not significantly improve biopsy-based fibrosis but reduced Pro-C3 levels (Loomba et al., 2023a). Nevertheless, a 240-week phase 3 trial (ESSENCE) in 1,200 adults with NASH and moderate to advanced liver fibrosis (stage 2 or 3) is currently underway, and the latest data from the first part of this trial from Novo Nordisk official website show that treatment with 2.4 mg semaglutide for 72 weeks significantly increased the proportion of patients who achieved improvements in liver fibrosis with no worsening of steatohepatitis (37.0% vs. 22.5%), as well as resolution of steatohepatitis with no worsening of liver fibrosis (62.9% vs. 34.1%) compared to placebo. This positive result is encouraging for NASH patients, and will be submitted for U.S. and EU regulatory approval in 2025. Furthermore, the combination of semaglutide with other drugs such as empagliflozin, cilofexor and firsocostat may enhance its effects on liver fibrosis (Lin et al., 2024; Alkhouri et al., 2022).

#### 3.1.2 GIP/GLP-1R agonist

Tirzepatide, a dual glucose-dependent insulinotropic polypeptide (GIP) and GLP-1 receptor agonist, promotes weight loss in patients with type 2 diabetes mellitus and obesity. In a phase 2 trial, 15 mg tirzepatide significantly decreased the levels of the fibrosis marker Pro-C3 in patients with type 2 diabetes mellitus compared to placebo after 26 weeks (Hartman et al., 2020). A recent phase 2 clinical study in patients with nonalcoholic steatohepatitis (NASH) and stage F2 or F3 fibrosis found that 52 weeks of tirzepatide administration significantly improved the resolution of NASH without worsening fibrosis, but had only a weak trend toward fibrosis regression (Loomba et al., 2024a). Due to the short duration and small sample size of this study, further studies are needed to investigate its effect on liver fibrosis (NCT05751720, NCT06374875).

#### 3.1.3 GCG/GLP-1R agonists

Survodutide (BI 456906) is a dual agonist of glucagon (GCG) and GLP-1 receptor that can inhibit the activation of HSCs and inflammation to mitigate liver fibrosis. In a phase 2 trial, survodutide significantly increased the proportion of participants received improvement in NASH without worsening of fibrosis, with a trend toward some degree of improvement in hepatic fibrosis as evidenced by a reduction in hepatic fibrosis obtained in 34% of the participants in the survodutide 2.4 mg group, 36% of those in the 4.8 mg group, 34% of those in the 6.0 mg group, and 22% of those in the placebo group (Sanyal A. J. et al., 2024). In people with compensated or decompensated cirrhosis, 28 weeks of survodutide treatment led to a trend of decreasing markers of liver fibrosis including liver stiffness, ELF scores, and plasma Pro-C3 (Lawitz et al., 2024). Moreover, the latest data from a phase 2 clinical trial conducted by Boehringer Ingelheim showed significant improvement in hepatic fibrosis after 48 weeks of administration with survodutide, as evidenced by the fact that 50.0% of the patients in the survodutide group achieved ≥1 stage improvement in fibrosis without worsening of NASH compared to 21.2% in the placebo group (Sanyal A. et al., 2024). These favorable results have prompted an ongoing phase 3 clinical trial to evaluate its effect on people with NASH/MASH and moderate or advanced liver fibrosis or cirrhosis (NCT06632457, NCT06632444).

#### 3.1.4 GCGR/GLP-1 agonists

Cotadutide, a dual GLP-1 and glucagon receptor (GCGR) agonist, has been reported to improve inflammation and fibrosis in NASH animals and this effect was stronger than that of liraglutide. In a 54-week randomized phase 2b study, in addition to lowering fatty liver index, cotadutide decreased FIB-4, NAFLD fibrosis score, and the PRO-C3 levels after 54 weeks of administration, and its effect on PRO-C3 was stronger than that of liraglutide (Nahra et al., 2021). Consistently, cotadutide also reduced these markers of hepatic fibrosis and fibrogenesis in participants with non-cirrhotic NASH with fibrosis (Shankar et al., 2024). Collectively, cotadutide can improve fibrosis, which may be stronger than GLP-1 receptor agonist liraglutide, and future evidence needs to be further consolidated by biopsy.

#### 3.1.5 Fibroblast growth factor 19 (FGF19) analogues

Fibroblast growth factor 19 (FGF19), an endocrine gastrointestinal hormone, suppresses CYP7A1 to regulate bile acid metabolism. FGF19 also modulates carbohydrate and energy metabolism. Aldafermin (also known as NGM282 or M70) is an engineered, nonmitogenic analog of FGF19 that has been developed for NASH. Initial studies demonstrated that 12 weeks of treatment with NGM282 could dose-dependently increase the proportion of patients with fibrosis improvement, accompanied by a decrease in noninvasive serum fibrosis biomarkers (Harrison et al., 2018a; Harrison et al., 2020a). A subsequent study reported a consistent trend in liver fibrosis changes after 24 weeks of administration (Harrison et al., 2021a). In the ALPINE 2/3 trial in patients with NASH and stage 2 or 3 fibrosis, there was no significant dose-response of aldafermin on the primary endpoint of histological fibrosis. However, a reduction in noninvasive markers was observed in the aldafermin group after 24 weeks of treatment (Harrison et al., 2022). The difficulty in improving the tissue levels may be attributed to the relatively short duration of drug administration. Recently, aldafermin has also been shown to reduce the enhanced liver fibrosis in patients with compensated NASH cirrhosis or primary sclerosing cholangitis (Hirschfield et al., 2019; Rinella et al., 2024). In brief, the antifibrotic effect of aldafermin is more sensitive to noninvasive detection. Notably, this drug has been associated with an increase in serum cholesterol levels (Rinella et al., 2019a), which may limit its further development.

#### 3.1.6 Fibroblast growth factor 21 (FGF21) analogues

Fibroblast growth factor 21 (FGF21) is a nonmitogenic hormone produced by the liver that regulates glucose and lipid metabolism and enhances secretion of adiponectin (Harrison et al., 2024a). Extensive efforts were made to modify the structure into engineered FGF21 analogues to extend the efficacy and half-life of FGF21 (Chui et al., 2024). Currently, there are several FGF21 analogues in clinical trials being developed for MASH remission and fibrosis improvement.

Pegbelfermin (PGBF), a polyethylene glycol-conjugated recombinant analog of human FGF21 with a circulating half-life of 19–24 h, was produced by the insertion of the novel amino acid p-acetyl phenylalanine (pAcF) at Q108, which serves as a designated conjugation site for PEG (Chui et al., 2024). It was initially found to improve metabolic parameters and fibrosis markers in patients with obesity and type 2 diabetes mellitus (Charles et al., 2019). Then, a phase 2a exploratory trial was conducted in NASH, and the results showed that 10 mg and 20 mg pegbelfermin were safe and able to reduce PRO-C3 levels (Sanyal et al., 2019). Subsequently, two phase 2b trials were launched to confirm its effect on NASH, publicly available data showed that patients with NASH and stage 3 (bridging) fibrosis or compensated cirrhosis did not achieve the histopathologic endpoint of fibrosis improvement without NASH worsening after treated with pegozafermin for 24 and 48 weeks (Loomba et al., 2024b; Abdelmalek et al., 2024). Due to this negative result, the development of pegbelfermin was discontinued.

Pegozafermin, a glycoPEGylated FGF21 analogue, was developed using a proprietary glycosyltransferase technology that allows site-specific linkage of a 20-kDa linear PEG to S173T via a glycosyl moiety to extend the circulating half-life to 2.5–4 days (Chui et al., 2024). It was shown to significantly reduce hepatic fat fraction and PRO-C3 in NASH patients with stage F1-F3 fibrosis in a phase 1b/2a study (Loomba et al., 2023b). A recent phase 2b trial found that treatment with 30 mg and 44 mg pegozafermin significantly led to 26% and 27% of patients with NASH and stage F2 or F3 fibrosis, respectively, achieving an improvement in fibrosis of at least one stage without worsening of NASH after 24 weeks, compared to only 7% for placebo (Loomba et al., 2023c). These data guide future trial designs, and two phase 3 studies are ongoing (NCT06419374, NCT06419374).

Efruxifermin,a human IgG1 Fc-FGF21 fusion protein with a circulating half-life of 3–3.5 days, was constructed by fusion of the Fc region of human IgG1 to a recombinant FGF21 variant with three point mutations (L98A, P171G, and A180E) to prevent protein aggregation and proteolytic cleavage, and to enhance the receptor-binding affinity (Chui et al., 2024). Administration of efruxifermin for 16-weeks significantly reduced liver fat and fibrosis markers including the enhanced liver fibrosis (ELF) scores and pro-C3 in a phase 2a trial (Harrison et al., 2021b). In a randomized controlled study of 30 patients with compensated NASH cirrhosis, the same results were obtained with 50 mg efruxifermin treatment for 16 weeks, interestingly, 4 out of 12 patients obtained an improvement in biopsy-based fibrosis (Harrison et al., 2023a). Moreover, 24-week administration of efruxifermin obviously improved hepatic fibrosis and NASH activity in patients with F2 or F3 fibrosis as demonstrated by 20% of patients in the placebo group having an improvement in fibrosis ≥1 stage and no worsening of NASH versus 39% of patients in the efruxifermin 28 mg group and 41% of patients in the efruxifermin 50 mg group (Harrison et al., 2023b). Currently, three phase 3 trials are underway to further evaluate the effects of efruxifermin in NASH (NCT06528314, NCT06215716, NCT06161571).

In short, the FGF21 analogues pegozafermin and efruxifermin are the few drugs reported to date that can improve biopsy-based fibrosis. Although the proportion of patients achieving improvement in liver fibrosis after 24 weeks of administration with efruxifermin appeared to be higher than that with pegozafermin (41% vs. 27%), there was a significant difference between the placebo group in the two trials (20% vs. 7%). The magnitude of placebo-adjusted improvement in fibrosis after efruxifermin treatment was similar to that of pegozafermin treatment for 24 weeks (21% vs. 20%), suggesting that the anti-hepatic fibrosis effects of efruxifermin and pegozafermin may be comparable. This assertion appears to be further supported by the similar half-life and FGFR agonism of these two drugs (Harrison et al., 2024a). However, recent meta-analyses mention that the efficacy of pegozafermin than that of efruxifermin (Zhong et al., 2025; Jeong et al., 2024). Therefore, which of the two drugs is stronger remains to be verified in head-to-head trials in larger populations. Moreover, attention should be paid to adverse gastrointestinal reactions such as nausea and diarrhea.

#### 3.1.7 Acetyl CoA carboxylase (ACC) inhibitor

During *de novo* lipogenesis, acetyl-CoA-carboxylase (ACC) and fatty acid synthase (FASN) convert metabolites of dietary sugars into the fatty acid palmitate, which is a key regulatory factor of lipid accumulation and lipotoxic substances, contributing to the pathogenesis of NASH (Loomba et al., 2021a). Antagonizing FASN or ACC not only reduces inflammation caused by a high-fat diet, but also blocks fibrosis by suppressing the activation of hepatic stellate cells, making FASN or ACC as potential targets for treating NASH (Wei et al., 2016; Bates et al., 2020).

Firsocostat (GS-0976, NDI-010976) is an inhibitor of acetyl-coenzyme A carboxylase (ACC) in the liver, and is intended to be developed for NASH. Following an open-label prospective trial in which GS-0976 was observed to obviously reduce liver stiffness and TIMP1 levels in patients with NASH compared to baseline (Lawitz et al., 2018), a randomized controlled trial was conducted in which, consistent with previous studies, 12-week administration of 20 mg GS-0976 significantly reduced liver fat levels and the fibrosis marker TIMP1, as well as lowered liver stiffness measured with the XL probe compared with placebo (Loomba et al., 2018a). Further studies in a 48-week phase 2b trial of advanced fibrosis due to NASH showed that firsocostat alone reduced noninvasive markers of fibrosis and steatosis. Moreover, firsocostat in combination with cilofexor further improved steatosis and fibrosis at multiple levels (Loomba et al., 2021b). This raises a new issue, namely, the risk of hypertriglyceridemia induced by this combination, which requires treatment with the lipid-lowering drug fenofibrate (Lawitz et al., 2023). In short, firsocostat has a certain anti-fibrotic effect, and its effect is stronger when used in combination with cilofexor, which supports further research.

#### 3.1.8 Fatty acid synthase (FASN) inhibitor

Denifanstat (TVB-2640) is a selective, potent, and reversible inhibitor of FASN currently under clinical development. In a phase 2 study (FASCINATE-1) in patients with NASH, treatment with denifanstat for 12 weeks resulted in a dose-dependent decrease in liver fat, liver biochemistry, and inflammation. Particularly, denifanstat significantly reduced the levels of fibrosis indicators such as TIMP-1, PRO-C3, and PIIINP (Loomba et al., 2021a). The latest data from a phase 2b study (FASCINATE-2) in patients with NASH and stage 2 to stage 3 fibrosis showed that the proportion of participants who achieved improvement of fibrosis by one stage or more without worsening of steatohepatitis or NAS by two points or more without worsening of fibrosis in the denifanstat group was significantly higher than that in the placebo group (41% vs. 18% or 38% vs. 16%) after 52 weeks of administration (Loomba et al., 2024c). These positive results support further investigations of the safety and efficacy of denifanstat in patients with MASH and F2/F3 fibrosis (NCT06594523).

#### 3.1.9 Stearoyl-CoA desaturase 1 (SCD1) inhibitor

Stearoyl-CoA desaturase 1 (SCD1) is a rate-limiting enzyme involved in monounsaturated fatty acid synthesis, fatty acid β-oxidation, and insulin sensitivity. A liver-targeted SCD1 inhibitor, aramchol downregulated the expression of fibrosis-related genes and proteins in hepatic stellate cells via inhibiting SCD1 and inducing PPARγ expression. In a phase 2b trial, 52 weeks of administration with 400 or 600 mg aramchol reduced the FIB4 score and NAFLD fibrosis score in patients with NASH. The latest data from the open-label part of the phase 3 trial showed that 300 mg Aramchol reduced hepatic fibrosis assessed using both conventional and digital pathology as well as noninvasive fibrosis tests, suggesting that Aramchol may represent a promising treatment for NASH and fibrosis, which warrants a phase 3 trial to evaluate its efficacy and safety (NCT04104321).

#### 3.1.10 Free fatty receptor agonist

Based on the fact that free fatty acid receptor (FFAR)1 and 4 (also known as GPR40 and GPR120, respectively) not only possess glycemic control effects by promoting GLP-1 production and inducing insulin secretion and sensitivity, but also have anti-inflammatory effects through inhibition of TAK1 (transforming growth factor-β-activated kinase 1) and the NLRP3 (NLR family pyrin domain containing 3) inflammasome, FFAR1/FFAR4 have been recognized as promising targets for diabetes and NASH treatment. Indeed, the semi-synthetic, eicosapentaenoic acid derivative icosabutate is an FFAR1/FFAR4 agonist, which was previously reported to inhibit inflammation as well as control blood glucose and liver enzymes in patients with hyperlipidaemia. Excitingly, a recent phase II clinical study confirmed that icosabutate significantly improved liver fibrosis in patients with MASH and F1-F3 fibrosis measured by both conventional and AI-assisted digital pathology, as manifested in that the proportion of patients with a ≥1-stage improvement in fibrosis in the icosabutate group was significantly higher than that in the placebo group (Harrison et al., 2025a). Of course, these results require larger clinical trials to further assess its anti-hepatic fibrosis efficacy and safety in patients with MASH.

#### 3.1.11 Thyroid hormone receptor-β (THR-β) analogs

The binding of thyroid hormones to their receptors (THR) plays an important role in maintaining normal physiological processes. THR is divided into two types: THR-α and THR-β. THR-β is mainly expressed in the liver, whereas THR-α is expressed in multiple organs, including heart, bone, and skeletal muscle. THR-β mediates lipid metabolism, inflammation and fibrosis, which are involved in NASH (Ratziu et al., 2024b). Resmetirom (MGL-3196) is a liver-directed THR-β-selective agonist developed for the treatment of NASH. The initial phase 2 clinical study explored in NASH patients that resmetirom not only significantly reduced liver fat levels, but also lowered the non-invasive fibrosis markers enhanced liver fibrosis and N-terminal type III collagen propeptide (PRO-C3), and 30% or more relative hepatic fat reduction in the resmetirom-treated group, which was associated with NASH resolution and a reduction in fibrosis stage on liver biopsy as well as improvement of quality of life (Younossi et al., 2022a; Harrison et al., 2019). An open extension study of this group of patients continued for 36 weeks after the completion of this study, and the continued administration of 80 and 100 mg resmetirom maintained improvement in fatty liver and liver fibrosis (Harrison et al., 2021c). For this reason, a phase 3 clinical study exploring 52 weeks of resmetirom administration showed safe and a reduction in hepatic fat, liver stiffness and TIMP-1 levels (Harrison et al., 2023c). A subsequent confirmatory phase 3 clinical trial in patients with NASH and fibrosis, unexpectedly and encouragingly, showed that NASH resolution with no worsening of fibrosis was achieved in 25.9% and 29.9% of the patients in the 80mg and 100 mg resmetirom group, respectively, as compared with 9.7% of those in the placebo group. Additionally, treatment with 80 mg and 100 mg resmetirom resulted in 24.2% and 25.9% of the patients achieving fibrosis improvement by at least one stage with no worsening of NAFLD activity, respectively, as compared with 14.2% of those in the placebo group (Harrison et al., 2024b). More importantly, these patients with NASH who experienced improvement in fibrosis or resolution of MASH after 52 weeks of treatment with resmetirom also had good improvements in quality of life (Younossi et al., 2024). Based on these positive results, resmetirom was the first drug approved by the U.S. Food and Drug Administration (FDA) for the treatment of NASH with moderate to advanced liver fibrosis in March 2024 (Keam, 2024). Currently, resmetirom is under regulatory review in the European Union. Several phase 3 trials are ongoing to further evaluate its effect on the treatment of NASH, including a pivotal serial liver biopsy/outcomes trial (NCT03900429), the supporting safety and biomarker trials (NCT04951219), and a second pivotal outcomes trial in participants with well-compensated NASH cirrhosis (NCT05500222) (Harrison et al., 2024c).

Given the importance of THR-β in NASH, other THR-β agonists such as VK2809 (NCT04173065), ALG-055009 (NCT06342947), HSK31679 (NCT05795517) have been developed, all of which are currently under clinical evaluation. Among them, HSK31679 has been reported to be superior to resmetirom in alleviating diet-induced steatohepatitis in preclinical models (Zhang et al., 2024).

#### 3.1.12 Peroxisome proliferator-activated receptor (PPAR) agonists

The peroxisome proliferator-activated receptors (PPARs) are nuclear receptors that mainly regulates metabolic homeostasis and inflammation (Staels et al., 2023). PPARs consist of three PPAR isotypes: α, δ (also called β), and γ. The differential distribution of these isotypes in tissues and cells determines their expression and activity. PPARs are involved in the development of fibrosis via regulating metabolism, inflammation and indirectly mediating HSCs activation (Gong et al., 2023).

##### 3.1.12.1 PPARα agonists

Fenofibrate is a PPAR-α agonist that can significantly lower liver stiffness and levels of hyaluronic acid (HA) and transforming growth factor beta 1 (TGF-β1) after 24 weeks of treatment in patients with NAFLD compared to before initiation of treatment. Moreover, the combination of pentoxifylline and fenofibrate could further reduce liver fibrosis (El-Haggar and Mostafa, 2015). However, fenofibrate did not reduce the liver fibrosis index in despite of a higher biochemical response in PBC (Liu et al., 2023).

Pemafibrate is a selective PPARα modulator that has been approved in Japan for the treatment of hypertriglyceridemia. Pemafibrate improved a variety of pathologies including steatosis and liver fibrosis in animal models of NASH. Treatment with 0.2 mg pemafibrate significantly reduced liver stiffness in patients with NAFLD lasting from 48 to 72 weeks, this response was associated with a reduction in the mac-2-binding protein glycosylation isomer, which is a novel liver fibrosis marker (Nakajima et al., 2021). Currently, two studies evaluating the efficacy of pemafibrate (K-808) in patients with PBC are recruiting (NCT06247735, NCT06247735).

##### 3.1.12.2 PPAR-γ agonists

Several evidences support that the PPAR-γ agonist pioglitazone effectively alleviates liver fibrosis caused by NASH, and is recommended for the treatment of NASH (Harrison et al., 2023d; Cusi et al., 2016). However, ineffectiveness in fibrosis and adverse effects such as weight gain have been sporadically reported (Sanyal et al., 2010). To reduce the side effects associated with PPAR-γ, a new chemical entity where deuterium modification of (R)-pioglitazone, PXL065 was developed. In a phase 2 study in patients with NASH, treatment with PXL065 for 36 weeks was observed to reduce procollagen type III and NAFLD fibrosis scores and improve fibrosis stage on histology (Harrison et al., 2023d).

Farglitazar, an insulin-sensitizing agent, selectively binds and activates PPAR-γ and has no effect on liver fibrosis in patients with chronic hepatitis C infection, as manifested by no differences in the levels of alpha-smooth muscle actin (SMA) expression and collagen as well as histologic assessments after 52 weeks of treatment (McHutchison et al., 2010).

##### 3.1.12.3 PPAR-δ agonist

Seladelpar is a potent and selective PPAR-δ agonist developed for the treatment of PBC. Multiple phase 3 studies have confirmed that seladelpar significantly improved liver biochemistry and pruritus (Hirschfield et al., 2023), but has no significant impact on liver stiffness or enhanced-liver-fibrosis scores (Hirschfield et al., 2024a).

##### 3.1.12.4 PPAR-α/γ dual agonists

Saroglitazar is a dual PPAR-α/γ agonist that has been approved for the treatment of NASH in India because of its beneficial effect in improving liver-related histology (Siddiqui et al., 2021). Two phase 2 studies observed that saroglitazar significantly reduced steatosis and weakly inhibited liver fibrosis (Siddiqui et al., 2021; Gawrieh et al., 2021). Preliminary studies have shown that saroglitazar also reduced ALP levels, however, its effect on liver fibrosis has not been studied in patients with primary biliary cholangitis (Vuppalanchi et al., 2022). At present, several randomized controlled studies are being conducted to evaluate the efficacy of saroglitazar in patients with PBC (NCT06427395, NCT05133336) or NASH and fibrosis (NCT05011305).

Aleglitazar is another potent dual PPAR-α/γ agonist that has been proven to significantly improve liver fibrosis and steatosis in patients with type 2 diabetes mellitus and coronary artery disease, characterized by a reduction in liver fat score, fibrosis-4, and NAFLD fibrosis score after 24 months of treatment (Grobbee et al., 2022). These results require further investigation of NAFLD.

##### 3.1.12.5 PPAR-α/δ dual agonist

Elafibranor (GFT505) is a dual PPARα/δ agonist approved by FDA in June 2024 for the treatment of PBC (Blair, 2024). Elafibranor is capable of decreasing hepatic lipid accumulation as well as downregulating pro-inflammatory and pro-fibrotic gene expression, thereby improving liver function in animal models of NAFLD/NASH and liver fibrosis (Staels et al., 2013). In a phase Ⅱ study in patients with NASH without cirrhosis, 120 mg elafibranor reduced the liver fibrosis stages in patients with NASH resolution (Ratziu et al., 2016), but there was no significant change in the proportion of patients with improvement in fibrosis without NASH worsening or resolution of NASH and improvement in fibrosis. Recent results from phase 2 or 3 clinical trials in primary biliary cholangitis have shown that elafibranor successfully improved biochemical indicators and disease activity markers (Schattenberg et al., 2021; Kowdley et al., 2024), but appeared to have little effect on liver fibrosis markers. In general, elafibranor may improve early lesions caused by different causes, such as steatosis, but has limited direct effects on fibrosis. However, its efficacy needs to be confirmed by the results of ongoing studies (NCT04526665, NCT06016842, NCT06383403, NCT06447168).

##### 3.1.12.6 Pan-PPAR agonists

Lanifibranor is a pan-PPAR agonist targeting α, β/γ, and δ, which can improve macrophage activation to reduce liver fibrosis and inflammation in preclinical models, and its efficacy is stronger than that of single or dual PPAR agonists (Sven et al., 2020). In a phase 2b trial in patients with noncirrhotic NASH, lanifibranor treatment for 24 weeks significantly promoted the regression of fibrosis, that is, 48%, 34%, and 29% of patients in the 1,200 mg or 800 mg lanifibranor or placebo group experienced improvement in fibrosis stage of at least 1 without worsening of NASH (Francque et al., 2021). Resolution of NASH and improvement in fibrosis stage of at least 1 was also observed in 35% of patients in the 1,200 mg lanifibranor group, 25% of patients in the 800 mg lanifibranor group, and 9% of patients in the placebo group in this study. Based on these findings, a phase 3 trial for assessment of lanifibranor is being conducted in patients with NASH and liver fibrosis stage F2 or F3 (NCT04849728).

Bezafibrate is a pan-PPAR agonist that has been shown to have potent anticholestatic efficacy in PBC patients with an incomplete biochemical response to ursodeoxycholic acid (UDCA) monotherapy (Honda et al., 2013). In a phase 3 trial conducted in patients with primary biliary cholangitis who had an inadequate response to UDCA, administration of 400 mg bezafibrate for 24 months reduced liver stiffness and enhanced liver fibrosis scores by 36% and 4% compared to placebo, respectively (Corpechot et al., 2018). Additional studies have confirmed that bezafibrate could improve moderate to severe pruritus in patients with primary sclerosing cholangitis and PBC (de Vries et al., 2021). Currently, several clinical trials are underway to evaluate the effects of bezafibrate alone or in combination with obeticholic acid in patients with PBC (NCT06443606, NCT06488911, NCT05239468, NCT04594694, NCT04514965).

#### 3.1.13 Farnesoid X receptor (FXR) agonists

The farnesoid X receptor (FXR) is an important member of the nuclear receptor superfamily, is mainly expressed in the liver, small intestine and kidney, and plays a key role in the metabolism of bile acids, glucose, and lipids. Multiple lines of evidence show that, in addition to regulating metabolism, FXR can participate in liver fibrosis by inhibiting inflammation and directly inhibiting the expression of ECM-related genes (Ding et al., 2024). FXR has emerged as an attractive target for drug development in the treatment of liver diseases, and over 10 FXR agonists have entered clinical phase II/III clinical development to evaluate their effects on PBC and NASH (Gioiello et al., 2024).

Obeticholic acid is a selective FXR agonist was approved for the treatment of PBC due to the positive results from a previous phase 3 study (Nevens et al., 2016). Short-term administration of obeticholic acid had no significant effect on noninvasive measures of liver fibrosis, but after 3 years of administration, obeticholic acid significantly improved fibrosis, and collagen morphometric features in this study (Bowlus et al., 2020). Consistently, two small-scale phase 2 studies in NASH observed that 25 mg obeticholic acid resulted in the reduction of the enhanced liver fibrosis score, improvement in steatosis, inflammation and fibrosis based on biopsy (Mudaliar et al., 2013; Neuschwander-Tetri et al., 2015). Moreover, a phase 3 clinical study including 931 patients with stage F1-F3 fibrosis further confirmed that 25 mg obeticholic acid significantly improved liver fibrosis and NASH disease activity as well as quality of life (Younossi et al., 2022b; Younossi et al., 2019). These data fully demonstrated the anti-fibrotic effect of obeticholic acid, but at the same time, drug-related pruritus and increased cholesterol occur in patients (Siddiqui et al., 2020), which to some extent limited its wide clinical application.

Cilofexor (GS-9674) is a potent and selective nonsteroidal FXR agonist that was developed to treat NASH and PSC. Cilofexor has demonstrated anti-fibrotic and anti-inflammatory activities in preclinical models. In a phase 2 study in PSC patients without cirrhosis, the authors found that 100 mg cilofexor administration for 12 weeks significantly improved biochemical indicators and showed a weak downward trend in liver fibrosis indicators TIMP-1 (Trauner et al., 2019), they continued to give cilofexor treatment for 96 weeks, and they found that cilofexor retained the improvement of liver biochemistry but increased the enhanced liver fibrosis score (Trauner et al., 2023). Similarly, another research group did not observe changes in liver fibrosis markers after 24 weeks of cilofexor treatment in patients with noncirrhotic NASH (Patel et al., 2020). All these results suggest that cilofexor alone has little effect on liver fibrosis, but its combination with firsocostat has recently been shown to induce the remission of liver fibrosis (Loomba et al., 2021b).

Other FXR agonists such as vonafexor and tropifexor have been shown to reduce liver fat content and liver enzyme levels (Ratziu et al., 2023; Anstee et al., 2023), but their effects on liver fibrosis require further verification.

### 3.2 Drugs targeting apoptotic signaling

#### 3.2.1 Antioxidants

Much of the literature suggests that oxidative stress is a key driver of hepatic steatosis and fibrosis. Reactive oxygen species (ROS) on the one hand directly activate HSCs to induce profibrogenic responses. On the other hand, it promotes hepatocyte death and the release of pro-inflammatory cytokines to exacerbate hepatic fibrogenesis (Blas-García and Apostolova, 2023). Therefore, antioxidants exerting hepatoprotective effects by antagonizing excessive oxidative stress have been recommended for the management of NAFLD. Vitamin E, a natural antioxidant, was recently confirmed in a multi-center, randomized, double-blind, placebo-controlled study to reduce liver fibrosis in MASH with 300 mg treatment for 96 weeks compared to the placebo group (Song et al., 2025). Although this study achieved statistical differences in histopathologic indicators of liver fibrosis, some clinical studies have observed that vitamin E is ineffective in liver fibrosis (Sanyal et al., 2010; Lavine et al., 2011), indicating that there is still a lack of strong data to support the benefit of vitamin E in ameliorating liver fibrosis. In preliminary trials, the combination of vitamin E and pentoxiphylline was found to be more efficacious than pentoxiphylline alone in obtaining fibrotic regression (Kedarisetty et al., 2021), suggesting that vitamin E may be more suitable as an adjuvant therapy for liver fibrosis.

NADPH oxidases (NOX) catalyze the production of superoxide and hydrogen peroxide. NOX enhances the proliferation and activation of HSCs and the production of inflammatory mediators, playing an important role in liver fibrogenesis (Blas-García and Apostolova, 2023). Setanaxib (GKT137831), a selective inhibitor of NOX1/4, has demonstrated to reduce ROS production, inflammation, and HSCs activation thereby attenuating liver fibrosis in preclinical models (Jiang et al., 2012). Setanaxib was observed to be safe in patients with primary biliary cholangitis and to have the potential to improve liver fibrosis in recent phase 2 clinical studies (Jones et al., 2023; Invernizzi et al., 2023), and these results support its further evaluation.

#### 3.2.2 Caspase inhibitors

Caspases are intracellular proteases that execute apoptosis and play an important role in inflammation and fibrosis. Emricasan is a pan-caspase inhibitor that has been shown to inhibit excessive apoptosis, resulting in anti-inflammatory and fibrotic effects in the liver of animal models. In several small population exploratory studies, emricasan was also able to reduce caspases and ALT levels in subjects with chronic hepatitis C or NASH (Shiffman et al., 2019). Treatment with 25 mg emricasan for 28 days reduced the hepatic vein pressure gradient and improved liver function in patients with compensated cirrhosis and severe portal hypertension (Garcia-Tsao et al., 2019). Subsequently, 3 months of treatment with emricasan was found to reduce INR and total bilirubin leading to improved MELD and Child-Pugh scores in patients with cirrhosis and end-stage liver disease (Frenette et al., 2019). However, in randomized placebo-controlled studies in patients with fibrosis or cirrhosis due to NASH, emricasan had a reduced effect on caspase-related biomarkers, but no significant effect on liver fibrosis and related death (Frenette et al., 2021; Harrison et al., 2020b; Garcia-Tsao et al., 2020).

#### 3.2.3 Inhibitor of apoptosis signal-regulating kinase 1 (ASK1)

Apoptosis signal-regulating kinase 1 (ASK1) is a serine/threonine signaling kinase that binds to thioredoxin to maintain homeostasis under normal physiological conditions. However, oxidative stress can dissociate ASK1 from oxidized thioredoxin, which in turn promotes the phosphorylation of p38 mitogen-activated kinase and c-Jun N-terminal kinase, ultimately causing a stress response that exacerbates apoptosis, inflammation, and fibrosis in the liver (Nelson et al., 2020). Inhibition of ASK1 has been reported to attenuate hepatic inflammation and fibrosis in animal models of NASH (Budas et al., 2016), demonstrating that ASK1 represents a potential target for NASH. Indeed, after 24 weeks of treatment with 18 mg and 6 mg of selonsertib, a selective inhibitor of ASK1, 43% and 30% of patients with nonalcoholic steatohepatitis and stage 2 or 3 liver fibrosis, respectively, experienced one or more stage reduction in liver fibrosis (Loomba et al., 2018b), compared with 20% of patients receiving an inactive therapy (simtuzumab). Interestingly, results from this phase II clinical trial showed that selonsertib-treated patients experienced remission of liver fibrosis associated with a decrease in liver stiffness, collagen content, inflammation, and apoptosis markers, and these patients also had a significant improvement in quality of life (Younossi et al., 2018; Jayakumar et al., 2019). However, treatment for 48 weeks with selonsertib did not alleviate fibrosis and liver-related clinical events in patients with bridging fibrosis or compensated cirrhosis due to NASH in subsequent phase III clinical trials, although it effectively inhibited hepatic p38 phosphorylation (Harrison et al., 2020c). The failure of this study may be because the fibrosis in the enrolled patients was too severe. In addition, the multiple pathways involved in the pathogenesis of advanced liver fibrosis may be one of the reasons why selonsertib is insufficient to reverse fibrosis. Nevertheless, a recent phase 2b trial showed that the combination of selonsertib with firsocostat or cilofexor for 48 weeks did not result in fibrosis regression at the levels of liver histology, imaging, and noninvasive markers (Loomba et al., 2021b), indicating that the anti-liver fibrosis effect of ASK1 inhibitors are not obvious in clinical practice.

### 3.3 Inflammatory and immune response modulators

Hepatic macrophages, also known as Kupffer cells, play a crucial role in the progression of liver fibrosis. Activated macrophages can trigger the production of inflammatory mediator and profibrotic molecules, such as TNF-α, IL-6, IL-1β, CCL2, TGF-β and PDGF. These mediators induce the activation of HSCs and the deposition of ECM to promote the development of liver fibrosis. Conversely, macrophages can also reverse or regress liver fibrosis by secreting IL-10 to induce apoptosis of activated HSCs and producing MMPs that degrade the ECM (Ran et al., 2025). Given the central role of macrophages in liver fibrosis, the development of macrophage-based antifibrotic drugs is increasing and showing well prospects. In addition to the targeting macrophage immune metabolism (ACC, PPAR, FXR) described above, there are many immunomodulatory drugs targeting macrophage-related molecules (CCR2/5, CCL24, galectin-3) described in this section.

#### 3.3.1 C-C chemokine receptor type 2 and 5 (CCR2/5) antagonist

Damaged hepatocytes stimulate macrophages to release C-C chemokine ligands 2 and 5 (CCL2 and CCL5), which bind to their receptor C-C chemokine receptor types 2 and 5 (CCR2 and CCR5), promoting the activation and migration of Kupffer cells and hepatic stellate cells, causing liver inflammation and fibrosis. Deletion of CCR2 or CCR5 obviously suppressed inflammatory cell activation and restored liver fibrosis in preclinical liver fibrosis models (Friedman et al., 2016). Likewise, as a dual antagonist of CCR2/CCR5, cenicriviroc (CVC) was observed to possess potent antifibrotic activity and a favorable safety profile in these models. Long-term and short-term administration of CVC also displayed excellent safety and tolerability in humans (Lefebvre et al., 2016; Francque et al., 2024). In the phase 2 CENTAUR study in patients with NASH and liver fibrosis, treatment with 150 mg CVC for 1 year resulted in twice the proportion of patients achieved improvement in fibrosis and no worsening of NASH compared to placebo (20% vs. 10%) (Friedman et al., 2018). Moreover, these effects were associated with a reduction in the levels of N-terminal type 3 collagen propeptide and enhanced liver fibrosis scores that could be maintained over 2 years, with stronger effects in patients with advanced fibrosis (Ratziu et al., 2020). However, the fibrosis-improving effect of CVC in NASH was not confirmed in the AURORA phase III study (Anstee et al., 2024). Since the anti-fibrotic effect of CVC is mainly mediated by the infiltration of macrophages, in fact, macrophages are highly heterogeneous and their phenotype can be reshaped according to different environments such as lipids, suggesting that other pathways may offset the anti-fibrotic activity of CVC.

Early studies found that CVC decreased soluble CD14 levels while increased CCL2 concentrations in HIV-infected adults (Thompson et al., 2016). This study also found that CVC could reduce liver fibrosis, as shown by a sustained decrease in the enhanced liver fibrosis test index and the fibrosis-4 scores (Friedman et al., 2016). Recent studies have reported that 200 mg CVC declined enhanced liver fibrosis index in HIV-1 infected patients after 48 weeks of treatment (Sherman et al., 2019). CVC also lowered the levels of plasma fibrotic biomarkers (transforming growth factor beta-1 [TGF-β1], thrombospondin-1 [TSP-1], and C-terminal pro-peptide of collagen type I [CICP]) in individuals living with HIV (Bowler et al., 2019). These findings suggested that CVC has the potential to restore HIV-induced liver fibrosis, but will need to be validated in future studies.

#### 3.3.2 C-C motif chemokine ligand 24 (CCL24) blocker

C-C motif chemokine ligand 24 (CCL24) is a chemokine produced by activated T cells, macrophages, and epithelial cells. CCL24 not only induces chemotaxis and activation of immune cells, but also stimulates the proliferation of human hepatic stellate cells and fibroblasts and collagen synthesis, playing an important role in inflammation and fibrosis through the C-C motif chemokine receptor 3 (CCR3) complex (Segal-Salto et al., 2020). Emerging evidences showed that CCL24 was elevated in the blood and liver of patients with NASH and primary sclerosing cholangitis, the levels of CCL24 were positively correlated with enhanced liver fibrosis scores. Interestingly, a humanized CCL24-neutralizing monoclonal antibody, CM101, was shown to significantly mitigate liver fibrosis and inflammation in preclinical models of NASH and primary sclerosing cholangitis (Segal-Salto et al., 2020; Greenman et al., 2023). Moreover, the first-in-human study confirmed that CM-101 has good safety and pharmacokinetic properties, and CM-101 can reduce the levels of inflammatory, fibrotic and collagen turnover biomarkers in patients with MASLD without evidence of MASH (Mor et al., 2024), supporting further investigation of CM-101 in the treatment of liver fibrosis. A phase 2a clinical trial to evaluate its safety and efficacy in subjects with primary sclerosing cholangitis is ongoing (NCT04595825).

#### 3.3.3 Phosphodiesterase (PDE) inhibitor

Pentoxifylline is a nonspecific PDE inhibitor with anti-inflammatory and antioxidant activities. Since previous clinical trials found that pentoxifylline could improve biochemical parameters and hepatic histological changes including inflammation and fibrosis (Zein et al., 2011; Zein et al., 2012), it was recommended by Japanese guidelines for the treatment of NASH patients in evidence-based clinical practice guidelines for nonalcoholic fatty liver disease/nonalcoholic steatohepatitis issued by the Japan Society of Hepatology in 2015 (Watanabe et al., 2015). However, the 2020 version of the guidelines did not include information on pentoxifylline. Perhaps its efficacy is not precise.

ZSP1601 is a first-in-class pan-phosphodiesterase inhibitor that can inhibit the secretion of tumor necrosis factor alpha (TNF-α) by elevating cAMP concentrations. ZSP1601 has been demonstrated to possess anti-inflammatory and anti-liver fibrosis effects in several preclinical animal models. The first-in-human study showed that ZSP1601 exhibited good tolerability and pharmacokinetic properties in healthy humans (Zhu et al., 2021). Moreover, a recent phase Ib/IIa trial showed that steatosis and fibrosis were effectively improved in patients with NAFLD after 28 days of treatment with ZSP1601, as evidenced by a reduction in liver chemistries, liver fat content, and FibroScan values compared to placebo (Hu et al., 2023; Li et al., 2024). These encouraging results have prompted further development of ZSP1601 for the treatment of NAFLD. Excitingly, a phase 2b study is underway to evaluate the efficacy and safety of ZSP1601 for 48 weeks in adult NASH patients (NCT05692492).

#### 3.3.4 Vascular adhesion protein-1 (VAP-1) blocker

Vascular adhesion protein-1 (VAP-1) is a sialoglycoprotein expressed on human hepatic endothelium that regulates lymphocyte adhesion and transendothelial migration. The levels of VAP-1 were significantly elevated in a variety of chronic diseases including NASH, chronic hepatitis B/C infection, and primary sclerosing cholangitis, their levels are associated with progressive liver fibrosis (Kraemer et al., 2019; Öksuz et al., 2020), representing a biomarker for monitoring the severity of fibrosis. Likewise, blockade of VAP-1 was observed to inhibit inflammatory cell infiltration into the liver and alleviate fibrosis in animals with liver injury (Weston et al., 2015). Surprisingly, no significant changes in inflammation and liver fibrosis were observed in patients with primary sclerosing cholangitis after treatment with anti-VAP-1 monoclonal antibody (timolumab, BTT1023) in a phase 2 clinical trial (Hirschfield et al., 2024b). Small sample size and short treatment duration may be partly responsible for these negative results. As VAP-1 is associated with other chronic liver diseases besides PSC, longer treatment durations and selection of appropriate outcome measures need to be considered in the future to evaluate the anti-fibrotic effect of this blocker.

#### 3.3.5 The leukotriene receptor antagonist

Leukotriene is generated by inflammatory cells, especially Kupffer cells in the liver, which bind to their receptor, cysteinyl leukotriene receptor 1(CysLTR1), to cause inflammation and fibrogenesis, while inhibition of CysLTR1 can restrain fibrosis-related indicators. Montelukast is a CysLT1 receptor antagonist approved for the treatment of asthma and allergic rhinitis. A recent preclinical study showed that montelukast could ameliorate carbon tetrachloride- and methionine-choline deficient diet-induced liver fibrosis through suppressing hepatic stellate cell activation and inflammation (Pu et al., 2023). This positive result inspired researchers to conduct a randomized double-blind placebo-controlled study in 52 patients with non-alcoholic steatohepatitis (NASH), patients were evaluated for liver stiffness and liver fibrosis biomarkers including hyaluronic acid (HA) and transforming growth factor beta-1 (TGF-β1) before and 12 weeks after treatment with montelukast or placebo, and showed that montelukast significantly reduced the values of liver stiffness measurement and levels of HA and TGF-β1 (Abdallah et al., 2021). The findings of this proof-of-concept study suggested that montelukast appears to a promising strategy for treating liver fibrosis in non-alcoholic steatohepatitis. However, its safety and efficacy against liver fibrosis need to be further validated in a larger population.

#### 3.3.6 Inhibitor of galectin-3

Galectin-3 is mainly secreted by macrophages and affects cell migration, adhesion, and inflammatory responses by binding to cell surface and extracellular matrix glycans. Galectin-3 is also able to regulate the activation of hepatic stellate cells and collagen production which participates in the development of liver fibrosis (Mackinnon et al., 2023). Available evidence supports that the increased galectin-3 levels in liver biopsies distinguished the F3/F4 from the F0/F1 fibrotic stages (Mackinnon et al., 2023). Accordingly, several galectin-3 inhibitors have shown anti-fibrotic effects in fibrotic disease models, among which representative agents are belapectin (GR-MD-02) and selvigaltin (GB1211). Clinical study results showed that belapectin was safe and well-tolerated for NASH patients (Harrison et al., 2016). However, patients with NASH and advanced fibrosis received 8 mg of belapectin for 4 weeks, which had no significant effect on inflammation and fibrosis (Harrison et al., 2018b). Additionally, subsequent studies found that belapectin reduced the hepatic venous pressure gradient and the development of varices in patients without esophageal varices, but it could not alleviate liver fibrosis in patients with NASH cirrhosis and portal hypertension after 52 weeks of administration (Chalasani et al., 2020). The inclusion of patients with severe fibrosis and cirrhosis, or inappropriate duration and dose of administration may have resulted in the failure of belapectin to improve liver fibrosis. Nevertheless, based on the positive results of subgroup analyses, the effect of belapectin on the prevention of esophageal varices in NASH cirrhosis is currently being explored (NCT04365868).

Selvigaltin (GB1211), derived from thiodigalactoside, has been shown to inhibit the expression of profibrotic genes in liver myofibroblasts and counteract liver fibrosis caused by CCl_4_ (Zetterberg et al., 2022). Selvigaltin was found to be well tolerated and safe in participants with hepatic impairment and in healthy participants (Aslanis et al., 2023; Aslanis et al., 2024). The developer then initiated a phase 2 clinical study to evaluate the effect of GB1211 in patients with NASH and liver fibrosis (NCT04607655), but the project was terminated in 2021 due to the impact of COVID-19 pandemic and changes in the clinical development strategy.

### 3.4 Drugs targeting the activation of hepatic stellate cells

#### 3.4.1 Targeting the TGF-β signaling pathway

TGF-β is a potent primary fibrogenic driver inducing the activation of hepatic stellate cells and other tissue myofibroblasts. TGF-β binds to its receptor, which contributes to fibrosis by activating SMAD-dependent canonical and multiple noncanonical pathways such as p38 MAPK, JINK, PI3K-Akt-mTOR, JAK, Rho-associated kinase (ROCK), etc. Therefore, TGF-β signaling appears to be an effective target for treating fibrosis (Li et al., 2021). Many inhibitors targeting TGF-β are currently under development. However, since TGF-β is a pleiotropic cytokine involved in cell proliferation and differentiation, immune regulation, cancer surveillance and wound healing, clinical trials were terminated due to toxicity in patients receiving antibodies or small molecules directly against TGF-β (Henderson et al., 2020). Currently, pharmaceutical developers are focusing on the downstream pathway of TGF-β to find new clues to solve the problem of fibrosis.

Pirfenidone is a drug that inhibits TGF-β, and has been approved by multiple national drug regulatory agencies for the treatment of idiopathic pulmonary fibrosis. A long time ago, a preliminary study showed that 30% of patients with hepatitis C virus chronic infection experienced a reduction in fibrosis after 12 months of treatment with pirfenidone (Armendáriz-Borunda et al., 2006). Many years later, they reported that treatment with 1,200 mg pirfenidone for 24 months resulted in a reduction in liver fibrosis in 67% of patients with chronic hepatitis C, and a decline in inflammation in 52% of these patients. Pirfenidone also decreased serum TGF-β1 and IL-6 levels as well as inhibited the expression of cannabinoid receptor CB2 in the liver (Flores-Contreras et al., 2014). To reduce adverse reactions, they recently used a prolonged-release formulation of pirfenidone to treat patients with advanced liver fibrosis and observed results consistent with those of previous studies (Poo et al., 2020), suggesting that pirfenidone represents a promising anti-fibrotic therapy for chronic liver diseases. Fortunately, this beneficial effect is being clinically verified (NCT05542615).

Hydronidone is a structurally modified drug derived from pirfenidone that is designed to reduce hepatotoxicity. It can inhibit liver fibrosis through the following two pathways: a) upregulating Smad7-mediated degradation of TGFβRI to inhibit activation of hepatic stellate cells (Xu et al., 2023), and b) inducing apoptosis of activated hepatic stellate cells through the endoplasmic reticulum stress-associated mitochondrial apoptotic pathway (Sun Z. et al., 2024). A phase 2 clinical trial in patients with chronic hepatitis B(CHB)-associated liver fibrosis showed that after 52 weeks of administration with hydronidone, 40.5%, 54.8%, and 43.9% of patients in the 180, 270, and 360 mg groups, respectively, achieved improvement in liver fibrosis, with the 270 mg group being significantly higher than that in the placebo group with 25.6% (Cai et al., 2023). These positive results have encouraged the initiation of two ongoing phase 3 clinical studies designed to evaluate the effectiveness of hydroxynidone in the regression of hepatic fibrosis in patients with HBV (NCT05905172, NCT05905172).

#### 3.4.2 Renin-angiotensin system blockers

Accumulating evidence suggests that angiotensin II (Ang II), the main peptide of the renin-angiotensin system (RAS), regulates the activation of HSCs, which result in fibrogenesis. Ang II induces the generation of TGF-β1 to cause the synthesis of matrix proteins. A retrospective study reported that patients with hepatitis C and hypertension who received angiotensin II blockade exhibited significantly less fibrosis (Corey et al., 2009). In addition, several preliminary studies in NASH and chronic hepatitis C observed that the angiotensin receptor antagonist, losartan reduced hepatic fibrosis, which was associated with reduced TGF-β1 and procollagen levels (Yokohama et al., 2004; Colmenero et al., 2009; Terui et al., 2002). Paradoxically, few studies have shown that angiotensin blocking agents are ineffective against fibrosis, which may be related to the heterogeneity of disease models and drug use (Hidaka et al., 2011; Abu Dayyeh et al., 2011). In this uncertain situation, randomized controlled studies in selected cirrhotic patients or compensated alcoholic liver fibrosis from different research groups demonstrated that candesartan, another angiotensin receptor blocking agent, not only lowered the hepatic venous pressure gradient, but also diminished liver fibrosis with a reduction in hyaluronic acid, TGF-β1 and extracellular matrix proteins (Kim et al., 2012; Debernardi-Venon et al., 2007). Consistently, the 6-month administration of candesartan or ramipril was recently found to significantly improve liver fibrosis in patients with chronic hepatitis C, with candesartan being more potent than ramipril (Mostafa et al., 2021). Together these results indicate that angiotensin receptor blocking agents including losartan and candesartan may represent a safe and effective therapeutic strategy for liver fibrosis.

#### 3.4.3 Integrin inhibitors

Integrins are the main cell adhesion receptors for the components of the ECM, regulating TGF-β activity and playing a central role in fibrosis. Several integrin inhibitors are being developed for antifibrotic therapy (Rahman et al., 2022), but only one drug, PLN-74809, a dual αvβ6/αvβ1 integrin inhibitor, is currently being evaluated in clinical trials for its effect on liver fibrosis in participants with primary sclerosing cholangitis and suspected liver fibrosis (NCT04480840).

#### 3.4.4 cAMP-response element-binding protein-binding protein (CBP)/β-catenin inhibitor

Under liver injury, the activated Wnt signaling prompts its downstream β-catenin to translocate into the nucleus, thereby recruiting CBP to induce target gene transcription, which plays an important role in the proliferation and activation of hepatic stellate cells. The inhibition of CBP/β-catenin has been reported to restore liver fibrosis by suppressing the activation of HSCs and increasing the production of matrix metalloproteinases (Osawa et al., 2015). In phase 1 or 1/2a clinical trials, OP-724 or PRI-724, a CBP/β-catenin inhibitor, has been confirmed to be safe and has potential anti-fibrotic effects in patients with hepatitis C and B virus-induced liver cirrhosis, as manifested by a significant reduction in liver stiffness and FIB-4 index after 12 weeks of administration (Kimura K. et al., 2022; Kimura et al., 2017). Several patients with advanced primary biliary cholangitis treated with OP-724 also showed improvements in fibrosis in a phase 1 study (Kimura M. et al., 2022). Importantly, OP-724 has recently been observed in patients with hemophilia combined with liver cirrhosis due to HIV/HCV coinfection not only to improve the liver stiffness measure and serum albumin levels, but also to reduce serum CXCL12 levels (Kimura et al., 2024). However, due to the small scale of these studies, their exact anti-fibrotic effects in patients with cirrhosis need to be further evaluated, which is currently ongoing (NCT06144086).

### 3.5 Drugs targeting extracellular matrix synthesis and degradation

#### 3.5.1 Knockdown of heat shock protein 47 (HSP47) with siRNA

Heat shock protein 47 (HSP47) is a collagen-specific chaperone residing in the endoplasmic reticulum that is essential for collagen synthesis (Ito and Nagata, 2017). Suppression of HSP47 was capable of reducing the generation of collagen and promoting the death of hepatic stellate cells to reverse fibrosis. BMS-986263 (ND-L02-s0201), a lipid nanoparticle delivering small interfering RNA designed to degrade HSP47 mRNA, has been developed into an siRNA therapeutic (Kavita et al., 2019). Previously, BMS-986263 has been revealed from a clinical phase 1b study to improve fibrosis at the histological level in patients with advanced fibrosis due to NASH or HCV. Consistently, results from a phase 2 study in patients with HCV infection and advanced fibrosis showed that BMS-986263 administration for 12 weeks also led to improvements in METAVIR and Ishak scores (Lawitz et al., 2022). However, a phase 2 study evaluating the efficacy and safety of BMS-986263 in adults with compensated cirrhosis from NASH was terminated due to a lack of short-term efficacy (NCT04267393). These findings hinted that BMS-986263 may be beneficial for patients with advanced liver fibrosis due to specific etiologies rather than cirrhosis, but this hypothesis still needs to be verified.

#### 3.5.2 Lysyl oxidase (LOX) inhibitors

Lysyl oxidase-like protein 2 (LOXL2) is a copper-dependent amine oxidase that catalyzes the cross-linking of collagen and elastin collagen and elastin, promoting stabilization of the extracellular matrix. Simtuzumab, a monoclonal humanized anti-LOXL2 antibody, was developed to prevent or reverse fibrosis (Chen et al., 2020). Although its anti-fibrotic effect was significant in preclinical liver fibrosis models, its efficacy in patients with bridging fibrosis or cirrhosis due to hepatitis C virus (HCV), human immunodeficiency virus (HIV), NASH, and primary sclerosing cholangitis (PSC) was negative when evaluated, showing no significant changes in liver histological and serum liver fibrosis markers after intervention with different doses of simtuzumab in the range of 75–700 mg (Harrison et al., 2018c; Meissner et al., 2016; Muir et al., 2019). A possible reason for this lack of benefit is the existence of alternative pathways that regulate collagen cross-linking, such as other LOX isoforms. Indeed, the pan-LOX inhibitor PXS-5505 (Yao et al., 2022) and the LOXL2/3 inhibitor PXS-5153A (Schilter et al., 2019) have been proven to have anti-hepatic fibrosis properties, among which PXS-5505 is currently undergoing clinical trials (NCT04676529), indicating that LOX is still a target worth developing to treat fibrosis.

## 4 Multi-target and pathway-guided anti-hepatic fibrosis therapy

### 4.1 Combination therapies

Although a few drugs have shown promising effects on liver fibrosis when used alone, most of them are ineffective or have adverse effects such as pruritus and hyperlipidemia. Due to the complex pathophysiology of liver fibrosis, targeting a single pathway along with the presence of complementary pathways results in drug therapy failure. Therefore, a combination therapy manipulating multiple pathways may be a viable therapeutic strategy for liver fibrosis. Indeed, in a phase 2b trial, the combination of cilofexor and firsocostat significantly reduced the ML NASH CRN fibrosis score, ELF score, and liver stiffness by transient elastography, whereas cilofexor or firsocostat alone had no significant effect on these fibrosis indicators (Loomba et al., 2021b). Similarly, the combination of pentoxifylline and fenofibrate resulted in more beneficial effects on HA, TGF-β, and liver stiffness in NASH patients than fenofibrate alone (El-Haggar and Mostafa, 2015). A phase 2 clinical study in patients with mild-to-moderate fibrosis due to NASH demonstrated that compared to monotherapy with semaglutide, combination therapy of semaglutide and cilofexor resulted in greater improvement in FAST score, although other non-invasive liver fibrosis markers such as liver stiffness by transient elastography and ELF scores showed no significant changes (Alkhouri et al., 2022).

Glucagon-like peptide-1 receptor agonists (GLP-1RAs) indirectly improved liver health by stimulating insulin secretion to regulate energy uptake. Semaglutide significantly reduced liver fat content and steatohepatitis but did not significantly improve hepatic fibrosis in patients with MASH and fibrosis stages 1–3 (F1-F3). Efruxifermin, a long-acting Fc-FGF21 analog that directly inhibits hepatic stress and collagen deposition, has been clinically reported to significantly ameliorate hepatic fibrosis and steatosis (Harrison et al., 2024a). The direct insulin-sensitizing effect of efruxifermin is thought to have complementary pharmacological effects with GLP-1RAs, which promotes insulin secretion. Indeed, a phase 2b study in adults with T2D and MASH with fibrosis (F1-F3) demonstrated that treatment with efruxifermin plus GLP-1RA for 12 weeks significantly reduced the levels of the markers of fibrosis (ELF score and FAST score) compared to GLP-1RA alone (changes from baseline over 12 weeks for ELF score, −0.6 vs. +0.1, P < 0.01. For FAST score, −0.16 vs. +0.04, P < 0.001) (Harrison et al., 2025b). These results conceptually validate the complementary pathway-based support for synergistic anti-hepatic fibrosis effects of the combination of efruxifermin and GLP-1RAs. In addition, a phase 2 clinical study enrolling 698 subjects to evaluate the effect of another FGF21 analog NNC0194 0499 in combination with semaglutide on liver injury and fibrosis in patients with NASH is ongoing (NCT05016882).

Recent animal studies have also confirmed that combining FGF21 agonism and CCR2/CCR5 inhibitor ameliorates steatohepatitis and fibrosis more potently than single-drug treatment (Puengel et al., 2022), which needs to be clinically verified. In addition to the synergistic effect, combined therapy can also reduce adverse reactions, such as rosuvastatin reducing the increase in serum cholesterol levels caused by the FGF19 analogue NGM282 (Rinella et al., 2019a). Unfortunately, several combinations of treatments such as tropifexor plus cenicriviroc, semaglutide plus firsocostat, selonsertib plus firsocostat have proven ineffective against fibrosis (Alkhouri et al., 2022; Loomba et al., 2021b; Anstee et al., 2023). In summary, the antifibrotic effect of the combination therapy was statistically significant but not strong. The observed success of the two-agent combination may be attributable to the use of more sensitive AI-driven assessment techniques and noninvasive fibrosis biomarkers for evaluation, whereas the conventional histological assessment, which is the gold standard, did not show strong results from the combination regimen. The lack of efficacy observed with the combination therapy may be twofold. Firstly, the limited sample size (e.g., 63 patients in the combination of cilofexor and firsocostat cohort compared to only 37/40 cases in the tropifexor plus cenicriviroc group (Loomba et al., 2021b; Anstee et al., 2023) likely resulted in an underpowered statistical analysis. Secondly, the multifactorial pathogenesis of the disease involving redundant pathways may explain this outcome. Notably, while semaglutide plus firsocostat showed no effect, the combination of the GLP-1 agonist semaglutide, FXR agonist cilofexor and ACC inhibitor firsocostat demonstrated a stronger reduction in the FAST score compared to semaglutide alone (Alkhouri et al., 2022).

### 4.2 Traditional Chinese medicine

Liver fibrosis is driven by multifactorial and multi-signaling pathways. Compared with the single target and limited effect of western drugs, Traditional Chinese Medicine (TCM) has been demonstrated as a potentially advantageous strategy for treating hepatic fibrosis due to its multi-target and pathway pharmacological effects (Liang et al., 2025). Interestingly, nearly 10 Chinese herbal compound prescriptions have been reported to have anti-fibrotic effects. The following section describes the latest clinical studies on four representative Chinese herbal compound prescriptions.

#### 4.2.1 Biejia-Ruangan compound (BRC)

The Biejia-Ruangan compound (BRC) is a traditional Chinese medicine that has been approved by the China Food and Drug Administration (CFDA) for treating liver fibrosis/cirrhosis caused by chronic hepatitis B (CHB). BRC exerts anti-fibrotic effects through the following multiple pathways (Li, 2020): inhibiting TGF-β/Smad-mediated fibrogenesis, restraining the proliferation and activation of HSCs, and enhancing the degradation of collagen. A multicenter, randomized, double-blind, placebo-controlled trial in 1000 CHB patients with advanced fibrosis or cirrhosis reported that after 72 weeks of treatment with entecavir plus BRC, the rate of fibrosis regression and cirrhosis reversal were significantly reduced, compared with the placebo group (Rong et al., 2022). After completing the 72-week trial, an open-label extension study was conducted on these subjects and found that entecavir plus BRC treatment could further reduce the risk of hepatocellular carcinoma and liver-related deaths (Ji et al., 2022). These results indicated that entecavir plus BRC is feasible for treating patients with CHB to reduce liver fibrosis and improve liver-related clinical outcomes.

#### 4.2.2 Fuzheng Huayu formula

Fuzheng Huayu formula (FZHY) is a compound formula consisting of 6 Chinese herbs, including Radix Salvia miltiorrhiza (Danshen), Persicae semen (Taoren), Cordyceps (Dongchongxiacao), Gynostemma pentaphylla (Jiaogulan), Schisandrae chinensis fructus (Wuweizi) and Pini pollen (Songhuafen). It was approved by CFDA for the treatment of delayed liver fibrosis back in 2002. Multiple clinical studies in Chronic Hepatitis B patients with liver fibrosis or cirrhosis have shown that FZHY in combination with nucleotide analogues including entecavir not only reduces noninvasive fibrosis markers but also improves Ishak fibrosis stages (Gui et al., 2020; Zhao Z. M. et al., 2022; Zhou et al., 2024). A retrospective study of 842 patients with hepatitis B-caused cirrhosis showed that FZHY combined with nucleotide analogues reduced the 5-year cumulative incidence of hepatocellular carcinoma (Shi et al., 2020). A recent phase 2b, randomized, placebo-controlled, double-blinded, multicenter study conducted in the United States also demonstrated that FZHY has significant anti-fibrotic effects in chronic hepatitis C patients with baseline Ishak F3 and F4 fibrosis stages (Hassanein et al., 2022). *In vitro* and *in vivo* studies revealed that FZHY exerts its antifibrotic effects comprehensively by integrating multiple pathways, as evidenced by not only inhibiting the activation of hepatic stellate cell and liver inflammation, protecting hepatocytes, but with novelty also inhibiting hepatic sinusoidal capillarization and angiogenesis (Zhou et al., 2024). In summary, these existing studies support the clinical application of FZHY in the treatment of liver fibrosis, particularly in hepatitis B-related liver fibrosis. However, evidence from studies in larger populations is still needed to re-validate its efficacy and safety.

#### 4.2.3 AnluoHuaxian pill (AHP)

The AnluoHuaxian pill (AHP) is an herbal formula approved for the treatment of liver disease. AHP can reverse liver fibrosis through inhibiting TGF-β1/Smad signaling pathway as well as balancing the synthesis and degradation of collagen. Data from randomized controlled studies showed that combined AHP and entecavir treatment for 78 weeks significantly elevated the improvement rate of hepatic fibrosis and suppressed the progression of liver fibrosis in patients with CHB (Miao et al., 2019). Not only that, monotherapy with AHP has also been demonstrated to improve liver fibrosis in CHB patients with normal or minimally elevated alanine transaminase levels and early liver fibrosis by histological assessment and noninvasive measurement after 48 weeks of treatment (Xiao et al., 2022), suggesting that AHP alone or in combination with antiviral drugs can resolve fibrosis, but its effect on liver-related clinical outcomes requires further study.

#### 4.2.4 Ruangan granule (RG)

Ruangan granule (RG) is a Chinese herbal formula that can alleviate blood stasis and dissipate hard masses. In a randomized controlled study that included 240 CHB patients with advanced liver fibrosis/early cirrhosis, analysis of liver-related changes in histopathology, serology, and imageology after 48 weeks of RG plus entecavir administration revealed that the combination of RG and entecavir treatment brought about improvements in hepatic fibrosis and inflammation, as well as further reductions in the risk of hepatocellular carcinoma at the 55-month follow-up, implying that RG can be used in combination with entecavir for the treatment of CHB (Xing et al., 2023).

## 5 Conclusion and prospects

Liver fibrosis is the “choke point” of chronic liver disease leading to hepatocellular carcinoma and liver failure. To date, the primary treatment is to eliminate the cause of the disease, however, many patients do not respond. In recent years, the development of anti-liver fibrosis drugs has become an area of great concern. The THR-β agonist resmetirom is the first drug approved by FDA to date to improve NASH-associated fibrosis, revolutionized the anti-hepatic fibrosis therapeutic landscape, and is currently being studied over a 52-month period for its impact on liver-related clinical outcomes. Although the failure of ASK1 and LOXL2 inhibitors, which were once thought to be promising, in phase 3 clinical studies were disappointing to developers, recent clinical trials have demonstrated that several candidates such as FGF21 analogues, FASN inhibitors, pan-PPAR agonists, GLP-1R agonists, GCG/GLP-1R agonists, hydronidone, etc. can improve biopsy-based hepatic fibrosis, which represents a new hope for antifibrotic therapy. Owing to the complex pathogenesis of hepatic fibrosis, multi-pathway-based combination therapy has been proposed as a new paradigm for attacking liver fibrosis, and the combination of cilofexor and firsocostat has a better effect on improving fibrosis than treatment alone. Importantly, Chinese medicines with multi-target and multi-pathway pharmacological activities have significant advantages in anti-hepatic fibrosis. In particular, BRC and RG not only improved liver fibrosis by histological assessment but also reduced liver-related clinical outcomes, which is the strongest anti-fibrotic effect observed to date and represents the state-of-the-art level of efficacy.

The approval of drugs by regulatory agencies requires proof of clinically significant benefits to patients, and in the United States and Europe, approval requires proof of improvement in the patient’s feelings, functions, and services. Since NASH is asymptomatic and takes several years to progress to cirrhosis, a major challenge in drug development is to develop and validate alternative indicators that predict a reduction in progression to liver-related outcomes. Based on the large body of evidence showing that liver fibrosis is strongly associated with the progression of NASH to end-stage liver disease such as cirrhosis, liver failure, liver cancer, and liver-related death, detection of changes in fibrosis is essential for assessing the benefits of NASH treatment. Currently, resolution of NASH without worsening of fibrosis and/or improvement in fibrosis without worsening of NASH are accepted as meaningful surrogate endpoints for accelerated or conditional approval in phase 3 trials in FDA and EMA (Cheung et al., 2019). Improvement in liver fibrosis greater than or equal to one stage obtained by drug treatment after excluding the placebo effect was considered an acceptable endpoint criterion, as demonstrated by multicenter, randomized, double-blind, placebo-controlled studies (Rinella et al., 2019b). The successful endpoint of a long-term confirmatory clinical trial should be a significant reduction in the risk of liver fibrosis-related death, cirrhosis, liver transplantation, and hepatocellular carcinoma with the drug. With the implementation of patient-focused drug development program, improvement in quality of life based on patient self-reporting is also being used as one of the criteria for assessing the anti-fibrotic effects of drugs (Harvey, 2022). Successful clinical studies of anti-hepatic fibrosis drugs require consideration of several elements, as follows: 1) Participant selection: the severity of the patient’s disease may affect the effectiveness of the drug. For example, selonsertib is effective in NASH with stage 2 or 3 liver fibrosis, but ineffective in patients with bridging fibrosis or compensated cirrhosis (Loomba et al., 2018b; Harrison et al., 2020c). 2) Sample size: Sample size is the key to determine the results of anti-fibrotic drug development. For example, in the phase 3 clinical study, the changes in the proportion of patients who achieved fibrosis improvement in obeticholic acid or resmetirom versus the placebo group were only about 10%, which was statistically significant. However, the changes in tirzepatide were more than 20%, but there was no statistical difference. The number of patients enrolled in the clinical trials of obeticholic acid, resmetirom, and tirzepatide was 931, 966, and 190, respectively (Loomba et al., 2024a; Harrison et al., 2024b; Younossi et al., 2019). 3) Dosage administered: the choice of dosage administered is critical to drug efficacy. For example, 15 mg pegozafermin had no significant effect on fibrosis improvement, while at 30 mg and 44 mg, the efficacy was statistically significant compared to placebo (Loomba et al., 2023c). Therefore, consideration should be given to the appropriate dosage range for administration during early clinical exploration to ensure that the drug is effective and safe. 4) Duration of administration: The progression of liver fibrosis has its own natural history, with untreated patients with primary biliary cholangitis advancing approximately one histological fibrosis stage every 1.5–2 years, with a 68%–82% probability of reaching an advanced liver disease stage after 4 years (Bowlus et al., 2020), therefore, clinical study design needs to take full account of the duration of administration. For example, short-term obeticholic acid had no effect, but administration of obeticholic acid for 3 years significantly reduced hepatic fibrosis (Bowlus et al., 2020). 5) Endpoint indicator selection, many drugs have obtained positive results in the exploratory study using noninvasive fibrosis markers as assays, whereas trials have failed when histologic assessment is the endpoint, suggesting that the reliability of imaging, histologic, and serologic markers should be comprehensively considered in the clinical evaluation process.

Liver biopsy is the gold standard for the diagnosis of liver fibrosis; however, its large-scale application is limited by invasiveness and sampling heterogeneity. Biomarkers are important guides for monitoring disease processes and evaluating drug responses to improve diagnostic and predictive sensitivity and reduce healthcare costs. Several markers such as ELF, NFS, APRI, FIB-4, FibroTest, and PRO-C3 have been reported to be associated with hepatic fibrosis staging and are used for disease stratification (Sanyal et al., 2023). These noninvasive fibrosis markers have also been used in the evaluation of drug efficacy, but there is insufficient evidence for these markers to identify early fibrosis and, as noted above, PRO-C3 is difficult to accurately identify the reliability of a drug in late-stage development (Reinson et al., 2023). Therefore, current noninvasive liver fibrosis markers require further verification. Simultaneously, with the advancement of multi-omics technology, more biomarkers of drug responses are yet to be discovered to accelerate the drug development process.

Currently, histological evaluation is performed by human pathologists, but reading variability and the lack of precise histologic definitions pose challenges. Recently, a measurement technique based on artificial intelligence has been developed for histopathological scoring, which has been compared in several drug clinical evaluations and shown to be consistent with expert pathologist consensus scores, with higher reproducibility and sensitivity (Ratziu et al., 2024a; Iyer et al., 2024; Ratziu et al., 2024c). Given these advantages, artificial intelligence-based histopathological scoring will be widely used in the evaluation of anti-fibrosis drugs in the future.

In conclusion, the clinical progress of anti-hepatic fibrosis drugs is gaining momentum. Appropriate clinical study design, identification of biomarkers, and advanced new drug evaluation technologies such as artificial intelligence and multi-omics will accelerate the clinical progress of anti-hepatic fibrosis drugs in the future.

## Glossary

ACC: Acetyl CoA carboxylase
AHP: AnluoHuaxian pill
Ang II: Angiotensin II
APRI: Aspartate aminotransferase-to-platelet ratio index
ASK1: Apoptosis signal-regulating kinase 1
BRC: Biejia-Ruangan compound
CBP: cAMP-response element-binding protein-binding protein
CCL2: C-C chemokine ligands 2
CCL24: C-C motif chemokine ligand 24
CCL5: C-C chemokine ligands 5
CCR3: C-C motif chemokine receptor 3
CFDA: China Food and Drug Administration
CHB: Chronic hepatitis B
CICP: C-terminal pro-peptide of collagen type I
CVC: Cenicriviroc
CXCL12: C-X-C motif ligand 12
CysLTR1: Cysteinyl leukotriene receptor 1
DAMPs: Damage-associated molecular patterns
ECM: Extracellular matrix
ELF: Enhanced liver fibrosis
EMA: European Medicines Agency
FASN: Fatty acid synthase
FDA: U.S. Food and Drug Administration
FFAR: Free fatty acid receptor
FGF19: Fibroblast growth factor 19
FGF21: Fibroblast growth factor 21
FIB-4: Fibrosis-4 index
FXR: Farnesoid X receptor
GCG: Glucagon
GCGR: Glucagon receptor
GIP: Glucose-dependent insulinotropic polypeptide
GLP-1R: Glucagon-like peptide 1 receptor
HA: Hyaluronic acid
HBV: Hepatitis B virus
HCV: Hepatitis C virus
HIV: Human immunodeficiency virus
HSC: Hepatic stellate cells
HSP47: Heat shock protein 47
IL-10: Interleukin-10
IL-1β: Interleukin-1β
IL-6: Interleukin-6
LOXL2: Lysyl oxidase 2
LSECs: Liver sinusoidal endothelial cells
MASH: Metabolic dysfunction-associated steatohepatitis
MASLD: Metabolic dysfunction-associated steatotic liver disease
MCP-1: Monocyte chemoattractant protein-1
ML: Machine learning
MMPs: Matrix metalloproteinases
NAFLD: Nonalcoholic fatty liver disease
NASH: Nonalcoholic steatohepatitis
NFS: Nonalcoholic Fatty Liver Disease Fibrosis Score
PBC: Primary biliary cholangitis
PDE: Phosphodiesterase
PDGF: Platelet-derived growth factor
PPAR: Peroxisome proliferator-activated receptor
PRO-C3: N-terminal type III collagen propeptide
PSC: Primary sclerosing cholangitis
RAS: Renin-angiotensin system
RG: Ruangan granule
ROCK: Rho-associated kinase
SCD1: Stearoyl-CoA desaturase 1
SGLT2: Sodium-glucose cotransporter-2
TCM: Traditional Chinese medicine
TGF-β: Transforming growth factor-β
THR-β: Thyroid hormone receptor-β
TIMP: Tissue inhibitor of metalloproteinase
TNF-α: Tumor necrosis factor-α
TSP-1: Thrombospondin-1
VAP-1: Vascular adhesion protein-1
